# Association mapping unravels the genetics controlling seedling drought stress tolerance in winter wheat

**DOI:** 10.3389/fpls.2023.1061845

**Published:** 2023-02-02

**Authors:** Matías Schierenbeck, Ahmad M. Alqudah, Samar G. Thabet, Ulrike Lohwasser, María Rosa Simón, Andreas Börner

**Affiliations:** ^1^ Genebank Department, Leibniz Institute of Plant Genetics and Crop Plant Research (IPK), OT Gatersleben, Seeland, Germany; ^2^ Cereals, Faculty of Agricultural Sciences and Forestry, National University of La Plata, La Plata, Argentina; ^3^ CONICET CCT La Plata, La Plata, Argentina; ^4^ Biological Science Program, Department of Biological and Environmental Sciences, College of Art and Science, Qatar University, Doha, Qatar; ^5^ Department of Botany, Faculty of Science, Fayoum University, Fayoum, Egypt

**Keywords:** drought tolerance, seedling, winter wheat, GWAS, candidate genes, PEG, FarmCPU, breeding

## Abstract

Drought is a major constraint in wheat (*Triticum aestivum L.*) grain yield. The present work aimed to identify quantitative trait nucleotides (QTNs)/ candidate genes influencing drought tolerance-related traits at the seedling stage in 261 accessions of a diverse winter wheat panel. Seeds from three consecutive years were exposed to polyethylene glycol 12% (PEG-6000) and a control treatment (distilled water). The Farm-CPU method was used for the association analysis with 17,093 polymorphic SNPs. PEG treatment reduced shoot length (SL) (-36.3%) and root length (RL) (-11.3%) compared with control treatments, while the coleoptile length (CL) was increased by 11% under drought conditions, suggesting that it might be considered as an indicator of stress-tolerance. Interestingly, we revealed 70 stable QTN across 17 chromosomes. Eight QTNs related to more than one trait were detected on chromosomes 1B, 2A (2), 2B, 2D, 4B, 7A, and 7B and located nearby or inside candidate genes within the linkage disequilibrium (LD) interval. For instance, the QTN on chromosome 2D is located inside the gene TraesCS2D02G133900 that controls the variation of CL_S and SL_C. The allelic variation at the candidate genes showed significant influence on the associated traits, demonstrating their role in controlling the natural variation of multi-traits of drought stress tolerance. The gene expression of these candidate genes under different stress conditions validates their biological role in stress tolerance. Our findings offer insight into understanding the genetic factors and diverse mechanisms in response to water shortage conditions that are important for wheat improvement and adaptation at early developmental stages.

## 1 Introduction

Globally, wheat is the main source of vegetable protein for humanity and provides around 20% of global calories for human consumption. It is considered a primary food staple in Europe, North Africa, Middle East and is growing in popularity in Asia (Food and Agriculture Organization, 2014). In wheat, Daryanto et al. (2016) reported that drought is a major yield-limiting factor worldwide, a situation that will increase in the future due to global climate change and uncertainties in precipitation patterns. Based on this problem, increasing the area sown in regions unsuitable for crop production due to early drought stress is one of the strategies for enhancing worldwide wheat production and meeting global demand (Thabet et al., 2018; Thabet et al., 2020). Faced with these scenarios, it is critical to identify the genetic factors determining different ecophysiological attributes related to abiotic stresses in order to accelerate the rate of genetic gain of the crop.

Due to the importance of rapid leaf area development, early seedling growth is a critical stage for wheat establishment (Ma et al., 2020; Maulana et al., 2020). Despite the great number of results achieved in basic and applied research, the genetic and physiological background of drought tolerance is still insufficiently known (Sallam et al., 2019; Thabet and Alqudah, 2019; Mahpara et al., 2022). Reporting candidate genes associated with drought tolerance traits and proving their functions is one of the major challenges facing genetics today and could become a key factor to increase global wheat production. The QTL (quantitative trait loci) mapping approach is a fundamental tool for the genetic dissection of agronomical traits (Muqaddasi et al., 2017; Alqudah et al., 2020; Schierenbeck et al., 2021), grain yield-related traits (Sukumaran et al., 2018), biotic stresses (Maccaferri et al., 2016) and abiotic stress tolerance (Landjeva et al., 2008; Lin et al., 2019). Plant response to polyethylene glycol (PEG) is comparable to that induced by drought in its early stages, causing a reduction in germination and seedling growth (Liu et al., 2006; Landjeva et al., 2008; Kido et al., 2016; Ayalew et al., 2018; Lin et al., 2019; Mahpara et al., 2022). Selection for higher coleoptile (Rebetzke et al., 2014; Ma et al., 2020), shoot (Reynolds et al., 2000; Landjeva et al., 2008) and root length (Ayalew et al., 2018) in early drought conditions has been reported as important strategies for increasing water shortage tolerance.

Recent studies reported several QTLs under drought conditions during post-flowering (Li et al., 2015; Sukumaran et al., 2018), while fewer efforts were documented for early crop stages under stress conditions (Maulana et al., 2020; Sallam et al., 2022). QTNs (quantitative trait nucleotides) or MTAs (marker-trait associations) related to coleoptile length on several chromosomes (Rebetzke et al., 2014; Ma et al., 2020), shoot length on 1A, 2B, 3A, 4B, 5A, 7A (Maulana et al., 2020; Maulana et al., 2021), root length on 2B, 2D, 3B, 4A, 4B, 5D, 5B and 6D (Kadam et al., 2012; Maccaferri et al., 2016; Ayalew et al., 2019; Xu et al., 2021) were documented previously. Moreover, Thabet et al. (2018) reported several significant SNPs associated with barley germination parameters and seedling related-traits when exposed to 20% PEG. Maulana et al. (2020) reported multiple significant QTN (quantitative trait nucleotides) related to early drought tolerance that was responsible for the shoot length growth under such abiotic conditions. Despite this, new efforts are necessary to evaluate different wheat accessions and environmental conditions to improve seedling drought tolerance as well as grain yield potential.

Several studies identified a number of promising candidates that had potential roles in drought stress tolerance in cereal plants, including wheat. In rice, high expression of *DEEPER ROOTING 1* (*DRO1*) increases the root angle and deep rooting enhancing grain yield under drought-affected soils (Uga et al., 2013). Consistent with the findings in rice, (Kulkarni et al., 2017) reported three copies of *DRO1* orthologs in the wheat genome. Overexpression of wheat Zinc Finger Proteins (ZFPs) in roots, *TaZFP34* gene, improved root-to-shoot ratio due to a decrease in shoot elongation while maintaining root growth during drought stress (Chang et al., 2016).

Therefore, the primary aim of the present study is to report MTAs controlling drought tolerance at the early growth stage in a recently described wheat panel constituted of 261 genotypes. For this purpose, the accessions were characterized for three years by the ability to maintain the root, coleoptiles, and shoot growth under PEG osmotic stress and further analyzed with 17,093 valid SNPs through a GWAS scan. Potential candidate genes controlling the studied traits under stress conditions had been detected and discussed in the current study. Our findings are a valuable resource for breeding new varieties carrying stress tolerance alleles and understanding the genetic mechanisms related.

## 2 Materials and methods

### 2.1 Plant phenotyping

To examine how large the natural variation in studied traits is related to seedling growth under control and induced drought conditions, a wheat panel comprising 261 accessions was tested. Detailed information about these panels was described by Schierenbeck et al. (2021).

Experiments were performed at IPK Gatersleben (Germany) using seeds grown under field conditions and harvested in 2016, 2017, and 2018. To induce drought stress (S), twenty seeds per accession were placed on filter paper humidified with PEG 6000 (12%) and grown in chamber conditions at 21°C in the dark for 3 days, and subsequently by 5 days at a 12-h photoperiod as suggested by Landjeva et al. (2008). Distilled water was used for control treatment [Control (C)]. After eight days, the shoot length (SL), coleoptile length (CL), root length (RL), and root/shoot length ratio (RSR) were recorded on ten seedlings per genotype. A tolerance index (TI) was defined for the four traits as the relation between the mean value obtained under stress and control conditions. In total, three replications were performed.

#### 2.1.1 Phenotypic data analysis

GenStat 19 software was used for the analysis of variance (ANOVA) and broad-sense heritability (*H^2^*) calculations:


$$H^{2}=\;\sigma g^{2}/\left(\frac{\sigma gy^{2}}{y}+\frac{\sigma e^{2}}{ry}\right)$$

where the mean squares for accessions (*σg^2^)*, genotype × environment interaction (*σgy^2^*), and residual error (*σe^2^)*, and *y* represents years and *r* (replicates). MVApp v2.0 (Julkowska et al., 2019) were used for correlations boxplot calculations.

The restricted maximum likelihood (REML) algorithm was applied and Best Linear Unbiased Estimators (BLUEs) for each treatment were calculated (BLUE-C and BLUE-S) using nlme package in R (Pinheiro et al., 2022).

### 2.2 Quantitative trait nucleotide analysis

#### 2.2.1 Genotyping and GWAS study

The panel was genotyped by Trait Genetics GmbH using the 90K iSELECT chip (Wang et al., 2014) generating 17,093 SNPs after filtering markers with >10% missing data and MAF <5%. Markers were mapped according to their physical position based on IWGSC RefSeq v1.1 (http://www.wheatgenome.org) and then used to determine the population structure, linkage disequilibrium (LD) and for GWAS scan as described in Schierenbeck et al. (2021). The genome-wide pairwise estimates of LD were calculated as a squared correlation between pairs of polymorphic SNPs (r^2^) for the whole genome using GenStat 18 (VSN International, 2015). Finally, LD decay patterns were visualized as the plot for the LD estimated the (r^2^) vs. the distance between pairs of polymorphic SNPs (Mbp) using R-package GGPLOT2 (Wickham, 2016). The average genome-wide LD decay at r^2^ = 0.2 (approximately 2.0 Mbp).

GWAS has performed by testing different statistical models in GAPIT 3 (R). The Farm-CPU model was chosen because it prevents model overfitting explained by a reduction in false-negative and false-positive associations (Bhatta et al., 2019; Schierenbeck et al., 2021; Wang & Zhang, 2021). The detected associations above the threshold of -log10 (p)≥3 in at least two environments were considered significant quantitative trait nucleotide (QTN) (Quan et al., 2021). A QTN was given to each significant SNP marker, and a quantitative nucleotide region (QNR) was given to the genomic regions (Gaur et al., 2022). For the highly associated QNR, LD block for specific physical distance including the strongest associated QTNs was calculated to define the physical interval of the associated QNR and then used for candidate gene detection (Alqudah et al., 2020).

#### 2.2.2 Candidate gene analysis

Those stable and highly significant QTNs were analyzed to detect those high-confidence candidate genes related, using RefSeq annotation v1.1 (http://www.wheatgenome.org). Gene Annotation description (GO and Interpros) was reported using the Wheat Mine platform (https://urgi.versailles.inra.fr/WheatMine/begin.do).

RNA‐Seq expression derived from the Wheat Expression Browser (http://www.wheat-expression.com/) was utilized for gene expression analysis of the multi-traits candidate genes. We also used the OPEN-ACCESS version of Genevestigator to check which gene is up or down expressed under drought and drought simulated by PEG from the expression and transcriptomes database (Hruz et al., 2008).

## 3 Results

### 3.1 Population structure and SNP coverage

As reported in our previous paper (Schierenbeck et al., 2021), the winter wheat panel clustered into three groups based on their continent of origin: 146 accessions derived from Eastern Europe-Western Asia (55.6%), 66 genotypes from Central-Northern Europe (25.2%) and 42 North-American accessions (16%). Regarding the SNP coverage, genome B showed the highest with 8,809 SNPs (51.5%), genome A was covered by 38.6% (6,595 SNPs) and D genome showed the lowest (9.9%) (Schierenbeck et al., 2021).

### 3.2 Phenotypic data analysis

The years, treatments, genotypes, *Treatments × Year* (except CL), and *Year × Treatments × Genotypes* interactions significantly influenced seedling growth parameters (**Table 1**). Drought treatment generates important reductions in growth parameters such as RL and SL, but an increase in CL and RSR. **Table S2** indicates a summary as well as the main results. For tolerance index, significant differences were reported in the four variables (**Table S1**).

**Table 1:** Means square and p-value (ANOVA) of coleoptile length, shoot length, root length and root:shoot length in an experiment with two drought treatments evaluated on 261 wheat genotypes during 3 years.

Source of variation	D.F.	Coleoptile Length (CL)	Shoot Length (SL)	Root length (RL)	Root/Shoot ratio (RSR)
***Years (Y)***	2	78.55 (<0.001)	171.6 (0.006)	157.3 (0.005)	11.0 (0.022)
**Error A**	4	0.56	7.42	6.28	0.97
***Treatment (T)***	1	138.4 (<0.001)	21907 (<0.001)	5272 (<0.001)	486.2 (<0.001)
**Y** *×* **T**	2	0.19 (0.268)	48.17 (<0.001)	59.2 (<0.001)	1.92 (0.029)
**Error B**	6	0.11	0.99	1.06	0.28
***Genotype (G)***	260	2.98 (<0.001)	22.93 (<0.001)	8.41 (<0.001)	1.09 (<0.001)
**Y** *×* **G**	516	0.11 (<0.001)	1.48 (<0.001)	1.58 (<0.001)	0.06 (<0.001)
**T** *×* **G**	260	0.12 (<0.001)	2.74 (<0.001)	1.21 (<0.001)	0.12 (<0.001)
**Y** *×* **T** *×* **G**	516	0.05 (<0.001)	0.68 (<0.001)	0.90 (<0.001)	0.039 (<0.001)
**Error C**	3104	0.023	0.22	0.41	0.01
**Total**	4673				
***H^2^***		0.98 (C) 0.97 (S)	0.97 (C) 0.94 (S)	0.89 (C) 0.87 (S)	0.98 (C) 0.95 (S)

H2, broad-sense heritability. ** indicate significance P<0.001; * P<0.05; ns (no significance) Control (C), Stress (S).

All variables analyzed under both growth conditions showed high broad-sense heritability ranging from 0.89 to 0.97 (control) and 0.87 to 0.97 under stress conditions (**Table 1**), while lower *H^2^* was detected for TI variables (**Table S1**).

**Figure 1:**
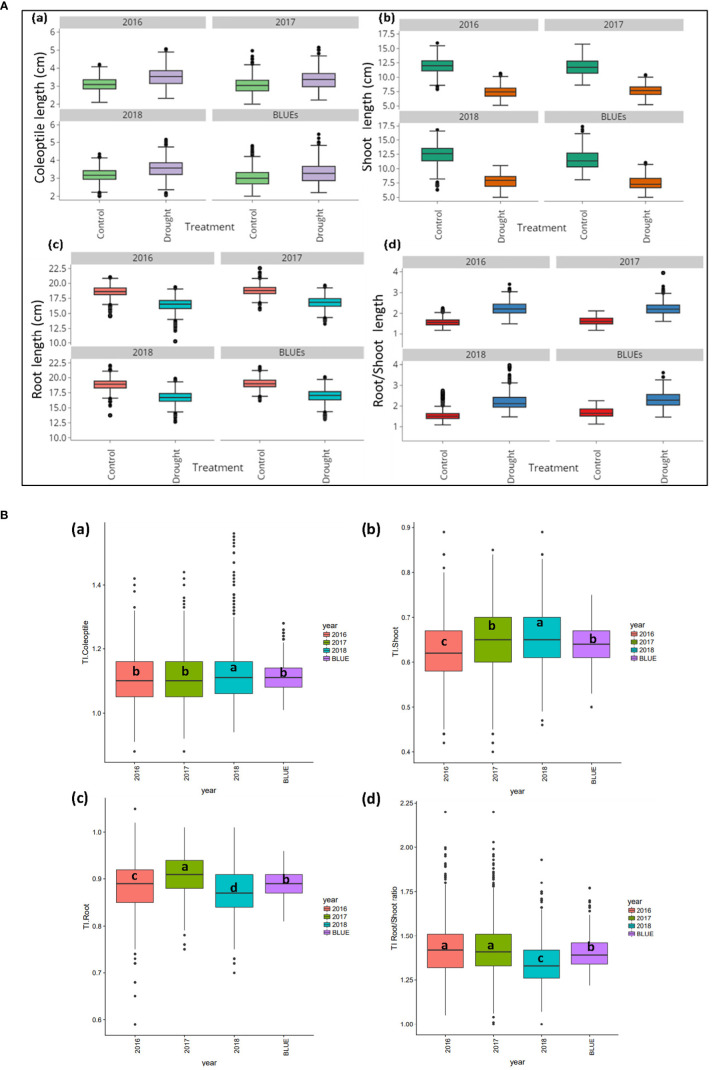
**(A)** Boxplot for real values for three years and BLUEs values for a) Coleoptile length (cm), b) Shoot length (cm) (c) Root length (cm), and d) Root/Shoot ratio in 261 winter wheat genotypes under control and drought stress. **(B)**. Boxplot for three years and BLUEs values for a) Coleoptile length tolerance index, b) Shoot length tolerance index c) Root length tolerance index and d) Root/Shoot ratio tolerance index in 261 winter wheat genotypes.

Compared to the control treatment, PEG treatments reduced SL by 36.3% and RL by 11.3% in wheat seedlings. In contrast, increases of 11% in CL were reported under drought. For CL, values across the three years varied from 2.00 to 4.47 cm in control conditions (BLUE-C) and 2.11 to 4.77 cm under drought stress (BLUE-S). The SL values ranged from 7.01 to 15.31 cm (BLUE-C) and 3.77 to 9.96 cm under PEG stress (BLUE-S). Variations in RL in control conditions fluctuated between 16.06 to 20.91 cm, while values from 13.79 to 19.50 cm were detected under drought conditions (BLUE-S). For RSR, BLUE-C values varied from 1.198 to 2.551 and 1.713 to 4.336 under drought stress. Variations of BLUEs values for CL, SL, RL, and RSR across years and summary statistics are indicated in **Table S2**. Variations among accessions across years under control and stress conditions as well as BLUEs values are shown in **Figure 1**.

**Figure 2:**
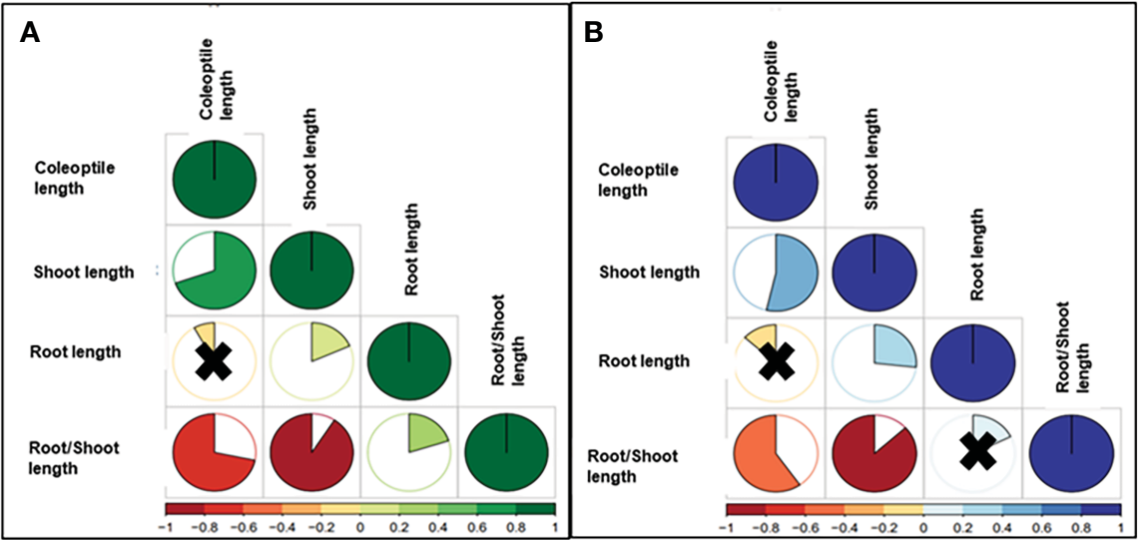
Pearson Correlation Coefficients of BLUEs values of the studied traits between **(A)** control and **(B)** drought in wheat genotypes. The degree of significance for all correlations was P<0.001. The color reflects the strength of the correlation. Black crosses indicate non-significant correlations.

Highly significant correlations between CL and SL were reported under control (0.71) and drought conditions (0.56). Negative correlations were reported between CL and RSR, showing values of -0.72 (control) and -0.61 (drought). Non-significant correlations were detected for CL and RL, -0.06 for control, and -0.12 under drought. The SL and RL presented positive correlations for control (0.18) and stress (0.27), while negative associations were reported for SL-RSR under control (-0.92) and stress experiments (-0.87) (**Figure 2**).

**Figure 3:**
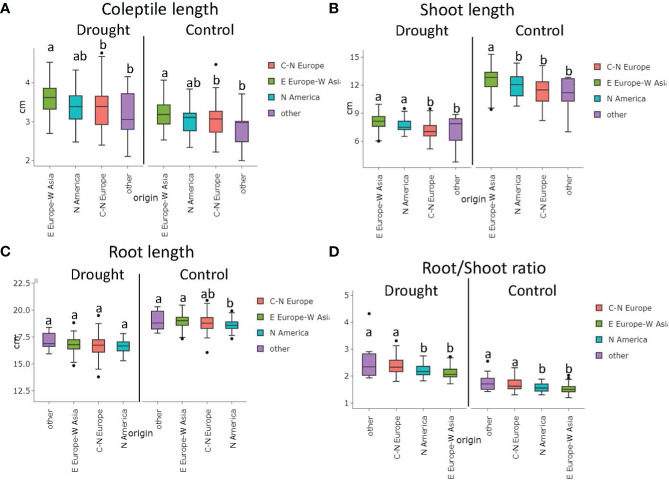
Variation on seedlings Coleoptile length **(A)**, Shoot length **(B)**, Root length **(C)**, and Root/Shoot Ratio **(D)** under control and drought stress based on genotype origin (BLUEs values). C-N Europe (Central-Northern Europe); E Europe- W Asia (Eastern Europe-Western Asia); N America (North America). Matching letters at thesame treatment are not statistically different (LSD p ≤ 0.05).

The diverse origins of the genotypes presented a differential response in the traits evaluated under control and stress conditions. Wheat accessions from Eastern Europe-Western Asia showed higher coleoptile and shoot lengths under control (3.19 cm and 12.63 cm, respectively) and stress conditions (3.57 cm and 8.08 cm, respectively) compared to those from Central and Northern Europe for CL (3.04 cm under control and 3.36 cm under stress) and SL (11.36 cm under control and 7.13 cm under stress). The North American cultivars showed intermediate values (**Figure 3**).

### 3.3 Genome-wide association mapping analysis

**Figure 4:**
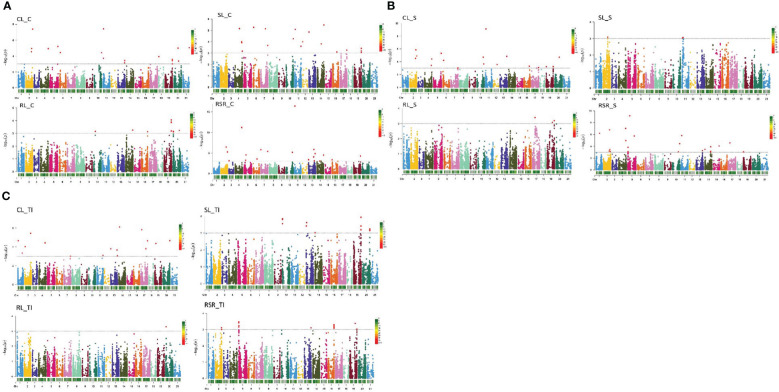
**(A)** Manhattan plots showing significant QTN (quantitative trait nucleotides) in 261 winter wheat genotypes used for seedling growth parameters (CL, SL, RL and RSR) under control conditions. Significant QTNs are indicated with red dots (P<0.001; -log10>3). **(B)** Manhattan plots showing significant QTN (quantitative trait nucleotides) in 261 winter wheat genotypes used for seedling growth parameters (CL, SL, RL and RSR) under stress conditions. Significant QTNs are indicated with red dots (P<0.001; -log10>3). **(C)** Manhattan plots showing significant QTN (quantitative trait nucleotides) in 261 winter wheat genotypes used for seedling growth tolerance index (CL, SL, RL and RSR). Significant QTNs are indicated with red dots (P<0.001; -log10>3).

The FARM-CPU model showed 70 stable QTNs in at least two environments (-log10>3; p<0.001) related to wheat seedling growth under control and drought stress across 17 chromosomes. These QTNs were reported on chromosomes 1A (1), 1B (6), 2A (4), 2B (9), 2D (2), 3A (2), 4A (1), 4B (3), 4D (2), 5A (9), 5B (2), 5D (1), 6A (2), 6B (4), 6D (1), 7A (13) and 7B (8) (**Figures 4A–C** and **Table S3**).

For CL under controlled conditions, ten QTNs were identified on 1B (1), 2A (1), 2B (1), 2D (1), 4B (2), 5A (1), 6B (1), 6D (1) and 7A (1) (**Figures 4A, B**). The most significant QTNs were IAAV971 on chromosome 4B, RAC875_c25839_225 on chromosome 6D and Kukri_c44587_130 on chromosome 1B. The phenotypic variation explained by QTNs (R^2^) ranged between 0.63 and 2.40% (**Table S3**). Under stress conditions (CL_S) seven, QTNs were found on 1B, 2A (2), 2D, 4B, 4D and 7A. Highly significant associations were reported for this trait in IAAV971 on 4B, IAAV6312 located on chromosome 2D and Kukri_c44587_130 on 1B. The R^2^ for this trait oscillated between 0.53 to 3.40%. For their part, one MTA was detected for CL_TI, BS00082219_51 on Chromosome 5A (**Figure 4C**).

Eleven QTNs were detected for RL under control conditions were localized on 5B, 7A (7) and 7B (3) (**Figures 4A, B**). BobWhite_c24096_57 on chromosome 7A, BS00089942_51 on chromosome 7B, and Tdurum_contig65330_190 on chromosome 5B. All showed a positive response to this trait (**Table S3**). The phenotypic variation explained by QTNs ranged between 0.08 and 3.27%. Under stress conditions, three stable QTNs were detected for RL: wsnp_Ra_c33358_42248399 on 6B, Kukri_c860_353 on 7A, and Kukri_rep_c72909_657 on 7B. R^2^ for the trait ranged between 0.48 and 2.07%. One QTN was related to the RL tolerance index (**Figure 4C**). Kukri_rep_c72909_657 and located on chromosome 7B.

Twenty-two QTNs were detected for SL (8 for SL_C, 2 for SL_S and 12 for SL_TI) (**Figures 4A–C**). For SL_C, QTNs were reported on chromosomes 2A (2), 4B, 4D, 5A, 6A, and 6B (2). QTNs such as wsnp_Ex_rep_c66270_64420584 on 2A, IAAV971 on 4B, wsnp_Ex_c3620_6612231 on 5A and BobWhite_c22827_193 on 6B showed the most significant associations with this trait (R^2^ between 0.6-2.16%). Under stress conditions (SL_S), wsnp_Ex_rep_c67036_65492436 on 1B and Kukri_c20822_1029 on 4B were documented. Twelve QTNs linked with the SL tolerance index were found on chromosomes 4A (1), 5A (5), 7A (3) and 7B (3). The most significant were Ex_c7626_444 on 4A, wsnp_JD_rep_c61843_39601402 on 5A, BS00061911_51 on 7A and wsnp_Ra_rep_c69221_66574260 on 5A. The phenotypic variation (R^2^) ranged between 0.65 and 4.06% (**Table S3**).

The RSR under control conditions showed 11 QTNs on 1B (2), 2A, 2B (2), 2D, 3A (2), 4B, 5A as well as 5B. The QTNs Kukri_c66451_128 on 1B, BS00060686_51 on 1B, BS00039422_51 on 2A, BobWhite_c2570_682 on 3A, Kukri_c18258_440 on 3A, *IAAV971* on 4B and Tdurum_contig17712_200 on 5A were the most significant ones. Under stress conditions (RSR_S), six QTNs were reported on chromosomes 1A, 2A, 2B (2), 4B and 5D. Highly significant associations were reported for GENE-4120_155 on 1A, RAC875_c52458_454 on 2A, BS00001140_51 on 2B; IAAV3303 on 2B and IAAV971 on 4B. Eight QTNs were detected for RSR_TI located on chromosomes 1B (2), 2B (5), and 6A (1). The most significant, such as tplb0034e07_1869, tplb0034e07_718, GENE-1442_78, Excalibur_c42248_663 and Kukri_rep_c108293_98 were reported on chromosome 2B (**Table S3**).

Moreover, eight multitraits QTNs across seven chromosomes were detected under control and stress treatments. These QTNs were detected on 1B (Kukri_c44587_130 related to CL-C and CL-S), 2A (RAC875_c52458_454 for CL-C/RSR-S/SL-C and RFL_Contig2656_871 for CL-C/CL-S), 2B (BS00001140_51 for RSR-C and RSR-S), 2D (IAAV6312 for CL_C and CL_S), 4B (IAAV971 for CL-C/CL-S/RSR-C/RSR-S/SL-C), 7A (BS00068033_51 for CL_S and SL_TI) and 7B (Kukri_rep_c72909_657 for RL_S and RL_TI) (**Tables 2**, **S3**).

**Table 2:** Distribution of pleiotropic loci or QTN (quantitative trait nucleotides) and candidate genes associated with two or more traits related to seedling growth under drought and control conditions.

Chr	Marker/Synonym	Trait/Effect/-LOG10 (p-value range	Marker Position (bp) and alleles	Candidate gene-Genomic location (bp)	Annotation (GO and InterPro)
**1B**	**Kukri_c44587_130** **(IWB45466)**	**CL_C (+0.066-0.094 cm)** **-LOG10 = 4.83-6.98** **CL_S (+0.091-0.132 cm)** **-LOG10 = 5.45-7.51**	**687795568.687795666** **(171,31 cM)** **A-G**	***TraesCS1B02G480400* (687794252-687799812)**	metal ion binding (molecular function) IPR036855 Zinc finger, CCCH-type superfamily
**2AL**	**RAC875_c52458_454** **(IWB58832)**	**CL_S (-0.064/-0.081 cm)** **-LOG10 = 3.57-4.24** **RSR_S (+0.047-0.092)** **-LOG10 = 3.20-6.02** **SL_C (-0.199/-0.233 cm)** **-LOG10 = 3.75-4.04**	**692755001.692755101** **(115,13 cM)** **C-T**	***TraesCS2A02G442700* (692754161-692757947)**	protein dimerization activity (molecular function) IPR036638 Helix-loop-helix DNA-binding domain superfamily
**2AL**	**RFL_Contig2656_871** **(IWB64018)**	**CL_C (-0.089/-0.104 cm)** **-LOG10 = 4.39-6.09** **CL_S (-0.098/-0.156)** **-LOG10 = 3.91-6.19**	**753540747.753540847** **(148,78 cM)** **A-G**	***TraesCS2A02G543900* (753538593-753541916)**	methionine biosynthetic process IPR011009 Protein kinase-like domain superfamily
**2B**	**BS00001140_51** **(IWB5784)**	**RSR_C (-0.049/-0.072)** **-LOG10 = 3.23-3.76** **RSR_S (-0.127/-0.221)** **-LOG10 = 3.62-9.64**	**183315442.183315542** **(95,82 cM)** **A-G**	***TraesCS2B02G203900* (183315109-183318519)**	IPR000644, CBS domain
**2D**	**IAAV6312** **(IWB35183)**	**CL_C (+0.055/0.063 cm)** **-LOG10 = 3.49-3.68** **CL_S (+0.064/0.184 cm)** **-LOG10 = 3.68-7.55**	**78765708.78765908** **(42,37 cM)** **A-C**	***TraesCS2D02G133900* (78764128-78767414)**	IPR000754 Ribosomal protein S9
**4BS**	**IAAV971** **(IWB35611)**	**CL_C (-0.143/-0.209 cm)** **-LOG10 = 10.71-12.48** **CL_S (-0.124/-0.249 cm)** **-LOG10 = 4.75-11.38** **RSR_C (+0.070/0.095)** **-LOG10 = 8.41-16.48** **RSR_S (+0.073/0.100)** **-LOG10 = 4.66-7.98** **SL_C (-0.374/-0.621 cm)** **-LOG10 = 5.66-7.38**	**40752368.40752568** **(57,48 cM)** **C-T**	***TraesCS4B02G051900* (40746325-40753688)**	protein kinase ATP binding IPR011009 Protein kinase-like domain superfamily
**7AL**	**BS00068033_51** **(IWB10213)**	**CL_S (+0.075-0.088 cm)** **-LOG10 = 3.37-4.20** **SL_TI (-0.012/-0.017)** **-LOG10 = 3.19-3.44**	**721417450.721417550** **(212,66 cM)** **A-C**	***TraesCS7A02G545300* (721411549-721417543)**	auxin response factor 2
**7B**	**Kukri_rep_c72909_657** **(IWB50233)**	**RL_S (+0.267-0.430 cm)** **-LOG10 = 3.05-4.09** **RL_TI (+0.009-0.016)** **-LOG10 = 3.27-3.82**	**46351467.46351567** **(53,75 cM)** **A-G**	***TraesCS7B02G047100* (46343243-46351992)**	IPR029005 LIM-domain binding protein/SEUSS

cM (centimorgan); Effect: Allelic effect in units (+- centimeters); Coleoptile Length (CL); Shoot Length (SL); Root length (RL); Root/Shoot ratio (RSR); C (control); S (Stress).

### 3.4 Candidate genes underlying wheat seedling growth under control and PEG stress

**Figure 5:**
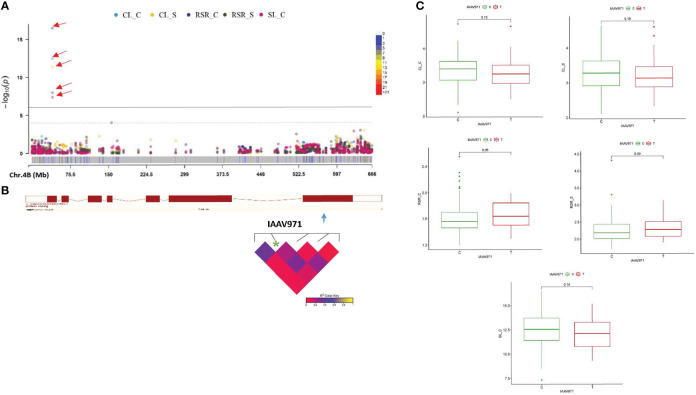
A Manhattan plots showing significant QTN (quantitative trait nucleotides) in 261 winter wheat genotypes for seedling growth parameters under (C) control and (S) drought stress **(A)** (CL_C, CL_S, RSR_C, RSR_S, and SL_C): CL, coleoptile length; RSR, root to shoot ratio; and SL, shoot length. Multitraits QTNs are indicated as (P<0.001; -log_10_> 3). **(B)** the structure of the TraesCS4B02G051900 gene with the position of the co-located QTN on 4B (40746325-40753688 bp) and the linkage disequilibrium (LD) interval, and **(C)** QTN -gene haplotype analysis.

Based upon GWAS outputs, we identified eight plausible candidate genes that are associated with two or more traits related to seedling growth under drought and control conditions. On chromosome 1B, the QTN Kukri_c44587_130 inside the *TraesCS1B02G480400* gene is located at a position 687794252-687799812 bp that harbor the CL phenotypic variation under both treatments. This CG is annotated as Zinc finger, CCCH-type superfamily that might be linked to biotic/abiotic stress tolerance in different species, including wheat (**Figures S1A, B**). For this QTN, accessions carrying the A allele showed longer coleoptiles under control and stress conditions (**Figure S1C**). The QTN RAC875_c52458_454 on chromosome 2A is located inside the gene *TraesCS2A02G442700* at position 692754161-692757947 bp and controls the variation of CL_S, RSR_S, and SL_C. Our candidate encodes a helix-loop-helix DNA-binding domain superfamily that pertain to the helix–loop–helix transcription factors (bHLH), playing a key part in regulating plant growth and development under various environmental stress conditions (**Figures S2A–C**). Genotypes carrying T alleles showed higher CL and SL under drought stress. A QTN (RFL_Contig2656_871) on chromosome 2A inside the candidate *TraesCS2A02G543900* at position (753538593-753541916 bp) controls the phenotypic variation of CL under both treatments and is annotated as a protein kinase-like domain superfamily (**Figures S3A, B**). The allelic effect for this QTN showed longer CL under both conditions for genotypes carrying the A allele (**Figure S3C**). The BS00001140_51 QTN was found inside the *TraesCS2B02G203900* gene (position 183315109-183318519 bp) on chromosome 2B associated to RSR under both control and drought conditions, and was annotated as CBS domain (**Figures S4A–C**). Another QTN (IAAV6312) on chromosome 2D is located within the *TraesCS2D02G133900* gene (position 78764128-78767414 bp) for CL under both conditions and encodes a ribosomal protein S9 that is involved in protein biosynthesis and regulates seed germination and seedling responses to drought stress (**Figures S5A, B**). For this QTN, genotypes carrying C allele showed longer coleoptiles under control and drought conditions (**Figure S5C**). Inside the *TraesCS4B02G051900* gene, a QTN (IAAV971) is located on chromosome 4B (position 40746325-40753688 bp) and controls the variation of CL, RSR under both treatments, and SL_C. This gene encodes the ATP-binding cassette (ABC) transporters mentioned as key contributors in diverse biological processes in different plant species, including phytohormones transportation, tolerance to a wide range of stresses as well as growth and development. (**Figures 5A** and **4B**). For this QTN, allelic effects showed longer SL_C and CL under both conditions, for genotypes carrying the C allele (**Figure 5C**). Markedly, the BS00068033_51 QTN on chromosome 7A is located within the candidate *TraesCS7A02G545300* (position 721411549-721417543 bp) for CL_S and SL_TI related-traits. This candidate is annotated as auxin response factor 2 which belongs to phytohormones that are involved in stem cell systems and meristems maintenance and development (**Figures S6A–C**). Finally, the QTN (Kukri_rep_c72909_657) on chromosome 7B within the *TraesCS7B02G047100* gene (position 46343243-46351992 bp) for RL_S and RL_TI was identified (**Figures S7A, B**). Moreover, accessions carrying the G allele showed a positive effect on drought tolerance for coleoptile and root length compared to accessions carrying the A allele (**Figure S7C**).

**Figure 6:**
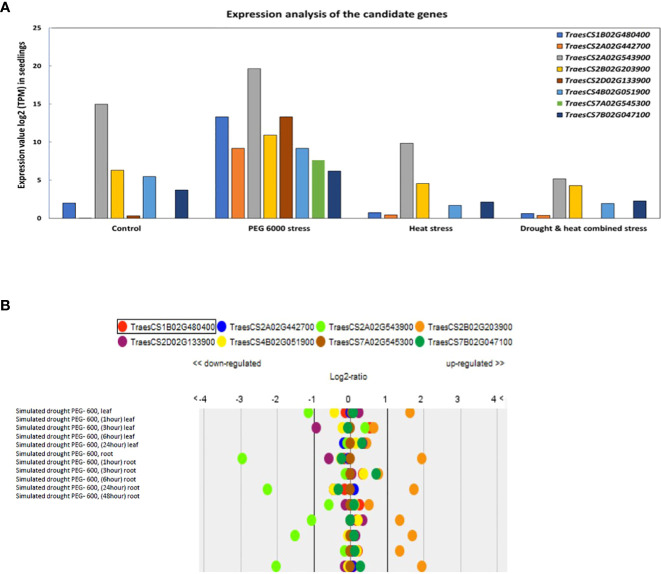
**(A)** Expression value log2 TPM (Transcripts Per Kilobase Million) of candidate genes in wheat seedlings after eight days under different stress conditions. **(B)** Up and down regulation under control and drought simulated by PEG from the expression and transcriptomes database.

The expression analysis of the eight multi-traits CG in contrasting growth conditions showed a wide range of gene expression. In that sense, all candidate genes presented a higher expression under 12%PEG compared with control and other stress conditions (**Figure 6A**). The genes *TraesCS2A02G543900, TraesCS2D02G133900*, and *TraesCS1B02G480400* showed the highest expression under drought stress PEG6000 while genes *TraesCS2A02G442700, TraesCS2B02G203900*, *TraesCS4B02G051900*, *TraesCS7A02G545300* and *TraesCS7B02G047100* showed an intermediate expression under the same conditions (**Figure 6A**). Based on the Genevestigator database, *TraesCS2B02G203900* was upregulated under simulated drought by PEG while *TraesCS2A02G543900* was downregulated in leaves and roots under PEG 6000 stress conditions (**Figure 6B**).

## 4 Discussion

### 4.1 Variability in seedling growth parameters

The detection of elements related to drought stress tolerance in the seedling stage is of major importance increasing the adaptation and accelerating the grain yield potential in environments where early droughts impose a primary constraint (Lin et al., 2019; Thabet and Alqudah, 2019). Despite the high number of identified QTN related to drought stress tolerance in post-flowering stages, studies regarding seedling growth-related traits under stress conditions are scarce or have been carried out only partially (Maulana et al., 2020). New efforts are necessary to explore worldwide wheat accessions across different conditions in order to identify the genetic basis of seedling drought tolerance and potentially enhance the MAS in breeding programs.

Our study explores the power of the FARM-CPU model to identify stable quantitative nucleotide regions (QNRs) associated with wheat seedling’s growth parameters in a recently reported wheat panel across three years. We reported extensive phenotypic variation as well as a high correlation among environments under stress and control conditions. The high heritability reported for all treatments (0.87–0.97) showed the utility of the set for breeding programs focused on drought stress tolerance in early crop stages as well as seedling growth habits for deep sowing. Consistent with our findings, Wei and Qi (1997) reported a highly significant correlation between CL and tolerance index under water shortage conditions, suggesting that coleoptile length might be used for drought-tolerance selection in early developmental stages in wheat breeding programs. For their part, Rauf et al. (2007) documented a significant and positive correlation between germination rate, CL, SL, and RL under control and PEG conditions. Moreover, the wide phenotypic variation of the panel provides more valuable inference in relation to Bi-parental populations, RILs, or DH lines due to a greater allelic diversity (Gupta et al., 2017; Sallam et al., 2019). Seedling´s growth parameters revealed significant discrepancies between genotypes origin. Higher CL, SL, and a lower RSR under both conditions were detected in Eastern Europe-Western Asia, while fewer differences were detected in root length.

### 4.2 QTN linked with seedling growth under PEG stress and control conditions

Our analysis reported QTNs related to drought stress tolerance in wheat seedlings across 17 chromosomes. These QTNs were stable across at least two environments, providing highly significant associations that can be used in further analysis.

Variations in CL from 2 to 4.47 cm under control and 2.11 to 4.77 cm under PEG stress conditions were documented. In total, 18 QTNs related to CL on chromosomes 1B (2), 2A (3), 2B, 2D (2), 4B (3), 4D, 5A, 6B, 6D, and 7A (2) were reported. Previous studies identified twelve significant QTLs on chromosomes 4B and 4D for coleoptile length in four wheat cultivars and explained 49% of the phenotypic variance (Rebetzke et al., 2007). Our findings agree with Yu and Bai (2010) that detected six significant QTLs for CL on chromosomes 1B, 3D, 4DS, 4DL, 5AS, and 5B, and some of them were also related to plant height in Chinese wheat landraces. Altogether, the colocalization of several QTLs across different chromosomes on the wheat genome suggested their potential roles in MAS programs.

Variations in SL from 7.01 to 15.31 cm (control) and 3.77 to 9.96 cm under PEG stress were reported. On chromosomes 1B, 2A (2), 4B (2), 4D, 5A, 6A, and 6B (2), ten QTNs related to SL were documented. Similarly, previous studies had reported some QTNs related to seedling growth parameters under early drought. For instance, five QTNs (*QSLDS.nri-2B, QSLDS.nri-3A*, and three *QSLDS.nri-4B*) were reported for shoot length at positions 153 cM, 68 cM, and 30-33 cM, respectively, as responsible for the variation of shoot length under drought condition (Maulana et al., 2020).

RL varied between 16.06 and 20.91 cm (control) and 13.79 to 19.50 cm under PEG conditions. Moreover, 14 QTNs were detected for RL under control and stress conditions. These markers were localized on chromosomes 5B, 6B, 7A (8), and 7B (4).In the same manner, Khalid et al. (2018) reported three QTN located on chromosome 7B between 122 to 128cM position, two related to root biomass (*QFRW.nust-7B*) that explained 59% of the phenotypic variance, and other for RL (*QRL.nust-7B*) under water-limited conditions. Moreover, Liu et al. (2012) explored nine QTNs for a doubled haploid wheat population related to the root morphological traits, including root length on chromosome 3B at the interval Xgwm644.2–P6901.2 under different water regimes. Using two related recombinant inbred line (RIL) populations in wheat, Zhang et al. (2013) detected two QTNs for root length under both control and drought stress located on chromosomes 1A, 4A, and 6B. A main-effect QTL from Weimai 8, with positive additive effects, was located between *Xswes131.3* and *Xswes131.4* on chromosome 6B under both water conditions.

We reported 17 QTNs associated with root/shoot ratio located on ten chromosomes (1A, 1B, 2A, 2B, 2D, 3A, 4B, 5A, 5B, and 5D). Genotypic variation for this trait ranged from 1.198 to 2.551 (control) and 1.713 to 4.336 under drought stress. Other authors reported QTLs related to this trait that increased RSR by reducing SL and maintaining RL during drought stress (Chang et al., 2016).

In total, 22 QTNs related to the tolerance index of parameters related to seedling growth were documented (1 for CL_TI, 12 for SL_TI, 1 for RL_TI, and 8 for RSR_TI) across seven chromosomes (1B, 2B, 4A, 5A, 6A, 7A, and 7B). Our results agree with Ayalew et al. (2017) who detected four root length QTLs on chromosome 1AS, 3AL, and 7BL with a total phenotypic variation of 47% under drought stress. Further, two root dry-weight QTLs with 10 and 15% of phenotypic variation were mapped to chromosomes 4 and 5AL under stress conditions. Taken these results together, Ayalew et al. (2017) reported that two major drought-responsive alleles were displayed in a diverse panel of wheat genotypes that caused long root length under PEG-induced drought stress.

### 4.3 Candidate genes underlying drought stress tolerance

Based upon GWAS outputs, we identified eight plausible candidate genes that are associated with two or more seedling growth parameters under early drought stress and control conditions as well as high gene expression under different stress conditions. On chromosome 1B, the QTN (Kukri_c44587_130) inside *TraesCS1B02G480400* influence the CL growth under controlled and 12%PEG stress conditions. This gene is annotated as CCCH-type Zinc (Zn) finger protein that belongs to zinc finger proteins which is involved in plant biotic and abiotic stress tolerance in various plant species. In wheat, the overexpression of *TaZnFP* has been associated with an enhancement in abiotic tolerance, particularly drought (Min et al., 2013). Our candidate provides a basis for exploring the molecular mechanisms by which zinc finger proteins play a specific role in drought tolerance.

The QTN (RAC875_c52458_454) on chromosome 2A is located inside the gene *TraesCS2A02G442700* and controls the variation of CL_S, RSR_S, and SL_C. Our candidate encodes the helix-loop-helix DNA-binding domain superfamily that plays a key role in growth and development under abiotic stress conditions (Duek and Fankhauser, 2005; Groszmann et al., 2010). In wheat, Liu et al. (2020) reported that the transcription factor TabHLH49 is related to the dehydrin *WZY2* gene expression and enhances drought stress resistance. Moreover, Wang et al. (2019) reported that the *TabHLH* expression levels were upregulated in response to drought stress conditions in multiple wheat organs.

On chromosome 2B, the BS00001140_51 marker was found inside the *TraesCS2B02G203900* gene for RSR under both control and drought, annotated as the CBS domain. This gene which encodes for CBS domain-containing proteins (CDCPs), was identified to be associated with tolerance to multiple plant stresses (Kumar et al., 2018). Moreover, Wang et al. (2010) detected that the CBS domain-containing protein gene *TaCDCP1* from wheat was upregulated and participated in the signal transmission pathways in response to drought stress. In rice under drought conditions, some CDCP genes (*OsCBSX9* and *OsCBSCBS4*) presented an increase in expression levels (Tomar et al., 2022). Understanding CBS domain signaling pathways are critical in breeding programs aimed at developing crop varieties more resistant to various environmental stresses, including drought.

Another QTN (IAAV6312) marker on chromosome 2D is located within the *TraesCS2D02G133900* gene for CL under both conditions and encodes as ribosomal protein S9. Previous reports detected an upregulation of the 40S and 60S ribosomal proteins in citrus when exposed to PEG-induced osmotic stress (Ziogas et al., 2015). In maize, most hub proteins such as 50S Ribosomal protein L2 (rpl2-A) were involved in protein biosynthesis through regulation of seed germination and seedling responses to drought stress (Yang et al., 2014). Therefore, it can be speculated that this gene is involved in drought stress responses, possibly through modulating plant growth and performance at the early developmental stage.

Inside the *TraesCS4B02G051900* gene, a QTN (IAAV971) located on chromosome 4B controls the variation of CL, RSR under both treatments, and SL_C. This gene encodes a ATP-binding cassette transporters (ABC) that is driven by ATP hydrolysis and acts as key contributors to diverse biological processes in different plant species, including growth, development, transport of phytohormones, stomatal closure, tolerance to biotic and abiotic stresses, etc (Dahuja et al., 2021). ABC transporters play a role *via* regulating the auxin and other phytohormones distribution inside the different organs and meristems (Lewis et al., 2007; Borghi et al., 2015). Under drought stress, Kuromori et al. (2014) and Krattinger et al. (2019) reported that the ABA transportation between the cytosol and the apoplast is regulated by the ABC transporters during the seed germination stage. For their part, *AtPDR12*/*ABCG40* ABC transporter play a role as ABA uptake transporter in the plasma membrane and further required for early stomatal closure during water-limited conditions (Kang et al., 2010). Our candidate gene can be considered to develop early drought-tolerant genotypes in wheat breeding programs.

Markedly, the BS00068033_51 marker on chromosome 7A is located within the candidate *TraesCS7A02G545300* for CL_S and SL_TI related-traits. This candidate is annotated as auxin response factor 2 which belongs to phytohormones that control the development and maintenance of plant meristems and stem cell systems (Friml et al., 2003; Kirschner et al., 2018). Phytohormones, including GA, cytokinin (Gordon et al., 2009), ethylene, ABA, brassinosteroids and strigolactones are key in the regulation of root and shoot differentiation (Marchant et al., 1999; Reinhardt et al., 2003). In maize (Ruzicka et al., 2009), rice (Yang et al., 2017) and *Arabidopsis* (Mussig et al., 2003), a reduction in RL and meristem size has been reported under high auxin concentrations. Thus, lower auxin concentrations are involved in regulating root growth in wheat (Kirschner et al., 2018). Altogether, this gene is a key regulator of root system architecture in wheat *via* modulating root length in response to drought stress conditions.

## 5 Conclusions

Via the novel FarmCPU method and using 17,093 valid SNPs, we reported 70 stable QTNs across 17 chromosomes associated with seedling growth parameters under control and drought stress conditions. Moreover, eight QTNs that are physically inside eight novel multi-traits candidate genes located on seven chromosomes showed high expression values in wheat seedlings under control and different stress conditions. Our findings offer insight into understanding the genetic factors and diverse mechanisms in response to water shortage conditions that are important for wheat improvement and adaptation at early developmental stages. New efforts should be made to explore the association between the CG functions with their effects on seedling growth under different sowing environments and dates as well as the effects of the genes on drought conditions imposed in later crop stages.

## Data availability statement

The original contributions presented in the study are included in the article/**Supplementary Material**. The genotypic data of 90K gene-associated SNPs used in this study was published in https://pubmed.ncbi.nlm.nih.gov/24646323/. Further inquiries can be directed to the corresponding authors.

## Author contributions

MS and AB designed the research. MS, ST, and AA analyzed data with help from MRS and UL. AB provided genotypic resources. MS, ST and AA wrote the manuscript with contributions from all co-authors. All authors contributed to the article and approved the submitted version.
