# Supplementary material for: Association mapping unravels the genetics controlling seedling drought stress tolerance in winter wheat
Source: Front Plant Sci. 2023 Feb 2;14:1061845. doi: 10.3389/fpls.2023.1061845 (PMC9933780; doi:10.3389/fpls.2023.1061845)
Supplement: Supplementary file 1 [file Table_1.docx]

**Table S1. Means square and p-value (ANOVA) of Coleoptile length Tolerance Index, Shoot length Tolerance Index, Root Length Tolerance Index and Root:Shoot Lenght Tolerance Index in an experiment with two drought treatments and 261 wheat genotypes evaluated during 3 years**

| **Source of variation** | **D.F.** | **Coleoptile Length TI (CL)** | **Shoot Length TI**  **(SL)** | **Root length TI**  **(RL)** | **Root/Shoot ratio TI**  **(RSR)** |
| --- | --- | --- | --- | --- | --- |
| ***Years (Y)*** | 2 | 0.083 (0.19ns) | 0.19 (0.046) | 0.29 (0.003) | 1.78 (0.041) |
| **Error A** | 4 | 0.032 | 0.026 | 0.008 | 0.23 |
| ***Genotype (G)*** | 260 | 0.022 (<0.001) | 0.018 (<0.001) | 0.007 (<0.001) | 0.077 (<0.001) |
| **Y × G** | 516 | 0.01 (<0.001) | 0.007 (<0.001) | 0.005 (<0.001) | 0.032 (<0.001) |
| **Error B** | 1552 | 0.003 | 0.0018 | 0.0012 | 0.009 |
| **Total** | 2336 |  |  |  |  |
| ***H^2^*** |  | 0.54 | 0.60 | 0.31 | 0.58 |

**Table S2. Summary statistics of Coleoptile length, Shoot length, Root length and Shoot to Root Ratio in an experiment with 261 wheat genotypes evaluated during 3 years**

| Trait | Year | Mean | Min. | Max. | Var. | Median | %CV | *s.d* |
| --- | --- | --- | --- | --- | --- | --- | --- | --- |
| *Coleoptile length*  *(cm)* | **2016c** | 3.348 | 1.92 | 4.97 | 0.185 | 3.36 | 12.84 | 0.430 |
|  | **2016d** | 3.693 | 2.12 | 5.45 | 0.287 | 3.74 | 14.50 | 0.535 |
|  | **2017c** | 2.965 | 1.92 | 4.55 | 0.151 | 2.96 | 13.08 | 0.388 |
|  | **2017d** | 3.285 | 1.80 | 5.15 | 0.255 | 3.27 | 15.38 | 0.505 |
|  | **2018c** | 2.967 | 1.80 | 4.44 | 0.131 | 2.98 | 12.22 | 0.363 |
|  | **2018d** | 3.329 | 2.04 | 5.07 | 0.227 | 3.33 | 14.32 | 0.477 |
|  | **BLUEc** | 3.095 | 2.00 | 4.473 | 0.132 | 3.127 | 11.74 | 0.363 |
|  | **BLUEd** | 3.438 | 2.106 | 4.77 | 0.212 | 3.483 | 13.39 | 0.460 |
| *Shoot Length*  *(cm)* | **2016c** | 12.42 | 7.27 | 17.40 | 3.048 | 12.52 | 14.06 | 1.746 |
|  | **2016d** | 7.697 | 3.12 | 11.05 | 1.527 | 7.68 | 16.05 | 1.236 |
|  | **2017c** | 11.45 | 6.34 | 14.65 | 1.871 | 11.46 | 11.95 | 1.368 |
|  | **2017d** | 7.352 | 2.92 | 10.52 | 1.252 | 7.36 | 15.22 | 1.119 |
|  | **2018c** | 11.85 | 6.30 | 15.51 | 2.035 | 11.89 | 12.04 | 1.427 |
|  | **2018d** | 7.713 | 3.34 | 10.65 | 1.182 | 7.69 | 14.10 | 1.087 |
|  | **BLUEc** | 11.91 | 7.01 | 15.31 | 1.928 | 12.05 | 11.66 | 1.389 |
|  | **BLUEd** | 7.589 | 3.77 | 9.96 | 0.917 | 7.586 | 12.62 | 0.958 |
| *Root length (cm)* | **2016c** | 18.54 | 14.54 | 22.07 | 1.238 | 18.63 | 6.00 | 1.112 |
|  | **2016d** | 16.33 | 10.24 | 19.64 | 1.583 | 16.43 | 7.71 | 1.258 |
|  | **2017c** | 18.71 | 15.63 | 21.58 | 0.732 | 18.70 | 4.57 | 0.856 |
|  | **2017d** | 17.05 | 13.17 | 19.73 | 0.974 | 17.12 | 5.80 | 0.987 |
|  | **2018c** | 19.27 | 13.74 | 22.55 | 0.784 | 19.24 | 4.60 | 0.886 |
|  | **2018d** | 16.82 | 12.98 | 20.14 | 1.408 | 16.82 | 7.05 | 1.187 |
|  | **BLUEc** | 18.84 | 16.06 | 20.91 | 0.445 | 18.84 | 3.54 | 0.667 |
|  | **BLUEd** | 16.72 | 13.79 | 19.50 | 0.621 | 16.75 | 4.71 | 0.788 |
| *Shoot/Root ratio* | **2016c** | 1.522 | 1.091 | 2.637 | 0.053 | 1.471 | 15.08 | 0.230 |
|  | **2016d** | 2.170 | 1.489 | 5.282 | 0.130 | 2.106 | 16.64 | 0.361 |
|  | **2017c** | 1.657 | 1.235 | 2.740 | 0.044 | 1.623 | 12.61 | 0.209 |
|  | **2017d** | 2.368 | 1.625 | 5.096 | 0.151 | 2.308 | 16.39 | 0.388 |
|  | **2018c** | 1.650 | 1.165 | 2.654 | 0.044 | 1.614 | 12.74 | 0.210 |
|  | **2018d** | 2.222 | 1.466 | 4.485 | 0.113 | 2.186 | 15.16 | 0.337 |
|  | **BLUEc** | 1.609 | 1.198 | 2.551 | 0.040 | 1.562 | 12.51 | 0.201 |
|  | **BLUEd** | 2.253 | 1.713 | 4.316 | 0.094 | 2.213 | 13.63 | 0.307 |

c (control), d (drought); Min.: Minimun; Max.: Maximun; Var.: Variance; %CV: coefficient of variation; s.d: standard deviation

**Table S3. Significant marker-trait associations and candidate genes for seedling stress tolerance traits in 261 winter wheat genotypes. Associations above the threshold of LOD≥3 in at least two environments were reported**

| Trait/Envoiroment | Marker/Synonym | Chr. | Position (bp) | R^2^ (%) | Effect | LOD | CANDIDATE GENE/POSITION (bp)/ANNOTATION |
| --- | --- | --- | --- | --- | --- | --- | --- |
| RSR_S_16  RSR_S_BLUE | GENE-4120_155  GENE-4120_155 | 1A | 9156772 | 0.66  0.63 | -0.083  -0.063 | 6.27  6.35 | **TraesCS1A02G017300.1** (9156307-9157322)  GO:0004364 GO:0005488 GO:0005829 GO:0009507 GO:0006749 GO:0009407 GO:0009651 GO:0009704 GO:0010304 GO:0010583 GO:0015824 GO:0046686 GO:0048527 GO:0048569 GO:0060416 GO:0080148 GO:0080167  Thioredoxin-like superfamily IPR036249  Glutathione S-transferase, C-terminal domain superfamily IPR036282 |
| SL_S_16  SL_S_BLUE | wsnp_Ex_rep_c67036_65492436  wsnp_Ex_rep_c67036_65492436 | 1B | 445432607 | 1.06  0.90 | 0.182  0.264 | 3.72  3.07 | **TraesCS1B02G252700** (445430105-445438995)  GO:0005543 GO:0046872 GO:0005886 GO:0005938 GO:0016021 GO:0009630 GO:0009887 GO:0009888 GO:0010413 GO:0010638 GO:0032065 GO:0033044 GO:0045492 GO:0051224  Regulator of chromosome condensation 1/beta-lactamase-inhibitor protein II IPR009091 |
| RSR_C_16 | Kukri_c66451_128 | 1B | 548902916 | 0.66 | 0.068 | 6.27 | **TraesCS1B02G558300LC** (548902104-548907590) scaffold88266 |
| RSR_C_BLUE | Kukri_c66451_128 | 1B | 548902916 | 0.20 | 0.060 | 6.42 |  |
| RSR_TI_17 | Kukri_c19247_53 | 1B | 614452510 | 3.96 | 0.037 | 3.35 | **TraesCS1B02G382000** (614451314-614456266)  GO:0004843 GO:0005488 GO:0006281 GO:0009058 GO:0048522 GO:0070536 GO:0070531 GO:0070552  BRCC36, C-terminal helical domain IPR040749  JAB1/MPN/MOV34 metalloenzyme domain IPR000555 |
| RSR_TI_BLUE | Kukri_c19247_53 | 1B | 614452510 | 2.06 | 0.025 | 3.08 |  |
| RSR_TI_17 | BobWhite_c48071_144 | 1B | 614455863 | 3.78 | 0.037 | 3.35 |  |
| RSR_TI_BLUE | BobWhite_c48071_144 | 1B | 614455863 | 2.06 | 0.025 | 3.08 |  |
| RSR_C_16 | BS00060686_51 | 1B | 675320327 | 1.21 | -0.040 | 5.48 | scaffold14272 (673204323-676151301) TraesCS1B02G771000LC |
| RSR_C_18 | BS00060686_51 | 1B | 675320327 | 0.75 | -0.037 | 5.89 |  |
| RSR_C_BLUE | BS00060686_51 | 1B | 675320327 | 0.80 | -0.031 | 5.19 |  |
| CL_C_17 | Kukri_c44587_130 | 1B | 687795617 | 0.87 | 0.094 | 6.98 | **TraesCS1B02G480400** (687794252-687799812) 8  GO:0046872 GO:0005634 GO:0005829 GO:0006355 GO:0006468 GO:0009059 GO:0016070 GO:0017148 GO:0030154 GO:0043488 GO:0044249 GO:0044282 GO:0048513 GO:0050896 GO:0051179  IPR036855 Zinc finger, CCCH-type superfamily |
| CL_C_18 | Kukri_c44587_130 | 1B | 687795617 | 2.25 | 0.063 | 4.83 |  |
| CL_C_BLUE | Kukri_c44587_130 | 1B | 687795617 | 1.25 | 0.066 | 5.00 |  |
| CL_S_16 | Kukri_c44587_130 | 1B | 687795617 | 1.24 | 0.132 | 7.51 |  |
| CL_S_17 | Kukri_c44587_130 | 1B | 687795617 | 0.53 | 0.100 | 5.45 |  |
| CL_S_18 | Kukri_c44587_130 | 1B | 687795617 | 2.51 | 0.091 | 6.42 |  |
| SL_C_16 | wsnp_Ex_rep_c66270_64420584 | 2A | 461979127 | 0.85 | -0.305 | 4.64 | scaffold36168 (437264744-462376174) |
| SL_C_BLUE | wsnp_Ex_rep_c66270_64420584 | 2A | 461979127 | 0.90 | -0.311 | 7.20 |  |
| CL_S_16 | RAC875_c52458_454 | 2A | 692755051 | 1.39 | -0.081 | 4.24 | **TraesCS2A02G442700** (692754161-692757947 )  GO:0003677 GO:0003700 GO:0046983 GO:0005634 GO:0006351 GO:0006355 GO:0009555 GO:0043068 GO:0048658 GO:0052543  IPR036638 Helix-loop-helix DNA-binding domain superfamily |
| CL_S_BLUE | RAC875_c52458_454 | 2A | 692755051 | 1.48 | -0.064 | 3.57 |  |
| RSR_S_16 | RAC875_c52458_454 | 2A | 692755051 | 0.29 | 0.047 | 4.34 |  |
| RSR_S_17 | RAC875_c52458_454 | 2A | 692755051 | 3.04 | 0.092 | 3.20 |  |
| RSR_S_BLUE | RAC875_c52458_454 | 2A | 692755051 | 0.47 | 0.062 | 6.02 |  |
| SL_C_16 | RAC875_c52458_454 | 2A | 692755051 | 1.23 | -0.239 | 4.04 |  |
| SL_C_BLUE | RAC875_c52458_454 | 2A | 692755051 | 1.00 | -0.199 | 3.75 |  |
| RSR_C_17 | BS00039422_51 | 2A | 693292855 | -0.20 | 0.053 | 8.43 | **TraesCS2A02G443400** (693291892-693298552 )  GO:0006139 GO:0006996 GO:0043170 GO:0050794 |
| RSR_C_18 | BS00039422_51 | 2A | 693292855 | 0.43 | 0.035 | 4.50 |  |
| RSR_C_BLUE | BS00039422_51 | 2A | 693292855 | 0.20 | 0.055 | 11.15 |  |
| CL_C_17 | RFL_Contig2656_871 | 2A | 753540797 | 0.92 | -0.089 | 4.39 | **TraesCS2A02G543900** (753538593-753541916)  GO:0000166 GO:0042802 GO:0046522 GO:0005634 GO:0005829 GO:0016020 GO:0006575 GO:0009309 GO:0016310 GO:0019284 GO:0019509 GO:0051790 GO:0065007 GO:0071281 GO:0071369 GO:0071732  IPR011009 Protein kinase-like domain superfamily |
| CL_C_BLUE | RFL_Contig2656_871 | 2A | 753540797 | 1.31 | -0.104 | 6.09 |  |
| CL_S_16 | RFL_Contig2656_871 | 2A | 753540797 | 1.48 | -0.156 | 6.19 |  |
| CL_S_17 | RFL_Contig2656_871 | 2A | 753540797 | 0.76 | -0.117 | 4.35 |  |
| CL_S_18 | RFL_Contig2656_871 | 2A | 753540797 | 2.69 | -0.117 | 4.63 |  |
| CL_S_BLUE | RFL_Contig2656_871 | 2A | 753540797 | 1.59 | -0.098 | 3.91 |  |
| RSR_TI_18 | tplb0034e07_1869 | 2B | 6311327 | 1.32 | -0.030 | 3.63 | **TraesCS2B02G012900** (6310652-6316308)  GO:0002943 GO:0055114 GO:0005829 GO:0034399 GO:0017150 GO:0046872 GO:0050660 |
| RSR_TI_BLUE | tplb0034e07_1869 | 2B | 6311327 | 2.12 | -0.024 | 3.07 |  |
| RSR_TI_18 | tplb0034e07_718 | 2B | 6313903 | 1.26 | 0.032 | 3.99 |  |
| RSR_TI_BLUE | tplb0034e07_718 | 2B | 6313903 | 2.30 | 0.026 | 3.31 |  |
| RSR_TI_18 | GENE-1442_78 | 2B | 6337735 | 1.32 | -0.030 | 3.63 | **TraesCS2B02G013000** (6336691-6341938)  GO:0000325 GO:0005774 GO:0005794 GO:0005886 GO:0016021 GO:0015334 GO:0071916 GO:0042937 GO:0006869 GO:0006891 GO:0010351 GO:0016558 GO:0042938 GO:0042939 GO:0050896  IPR036259 MFS transporter superfamily  IPR000109 Proton-dependent oligopeptide transporter family |
| RSR_TI_BLUE | GENE-1442_78 | 2B | 6337735 | 2.12 | -0.024 | 3.07 |  |
| RSR_TI_18 | Excalibur_c42248_663 | 2B | 6715738 | 1.36 | 0.032 | 3.79 | **TraesCS2B02G013500** (6710935-6716133)  GO:0005886 GO:0007275 GO:0044237 GO:0044238 GO:0050794 GO:0050896  CYTOMATRIX PROTEIN-LIKE PROTEIN (PTHR35992) |
| RSR_TI_BLUE | Excalibur_c42248_663 | 2B | 6715738 | 2.07 | 0.027 | 3.37 |  |
| RSR_TI_18 | Kukri_rep_c108293_98 | 2B | 7443104 | 1.10 | -0.030 | 3.59 | **TraesCS2B02G015500** (7428456-7443833)  Biological Process: histone H3-K36 methylation (GO:0010452)  GO:0005634 GO:0006351 GO:0006730 GO:0009553 GO:0009555 GO:0009910 GO:0010223 GO:0010363 GO:0010452 GO:0016116 GO:0019219 GO:0031062 GO:0040029 GO:0043687 GO:0048481 GO:0048653 GO:0051276 GO:0008270 GO:0042800 GO:0046975  IPR011124 Zinc finger, CW-type; IPR001214 SET domain; IPR006560 AWS domain |
| RSR_TI_BLUE | Kukri_rep_c108293_98 | 2B | 7443104 | 2.02 | -0.025 | 3.14 |  |
| RSR_C_16 | BS00001140_51 | 2B | 183315492 | 0.46 | -0.072 | 3.76 | **TraesCS2B02G203900** (183315109-183318519)  GO:0005975 GO:0006633 GO:0016310 GO:0042128 GO:0043170 GO:0050794 GO:0050896 GO:0080090 GO:0009505 GO:0016021 GO:0016301  IPR000644 CBS domain |
| RSR_C_BLUE | BS00001140_51 | 2B | 183315492 | -0.05 | -0.049 | 3.23 |  |
| RSR_S_16 | BS00001140_51 | 2B | 183315492 | 0.57 | -0.183 | 9.94 |  |
| RSR_S_17 | BS00001140_51 | 2B | 183315492 | 2.80 | -0.221 | 3.62 |  |
| RSR_S_BLUE | BS00001140_51 | 2B | 183315492 | 0.50 | -0.127 | 6.57 |  |
| RSR_C_17 | Tdurum_contig50509_805 | 2B | 216467652 | 0.91 | 0.028 | 4.05 | **TraesCS2B02G225800** (216448836-216468232)  GO:0005515 GO:0004222 GO:0008270 GO:0005615 GO:0005654 GO:0005739 GO:0005777 GO:0005829 GO:0009986 GO:0006508 GO:0010815 GO:0032461 GO:0032501 GO:0044260 GO:0008152 GO:0051260  IPR007863 Peptidase M16, C-terminal |
| RSR_C_BLUE | Tdurum_contig50509_805 | 2B | 216467652 | 0.90 | 0.026 | 4.43 |  |
| RSR_S_16 | IAAV3303 | 2B | 615365740 | 2.08 | -0.098 | 3.68 | **TraesCS2B02G428100** (615364743-615367066 )  GO:0005737 GO:0016020 GO:0006139 GO:0009058 GO:0044260 GO:0044281 GO:0050896 GO:0016791  IPR023198 Phosphoglycolate phosphatase-like, domain 2 |
| RSR_S_BLUE | IAAV3303 | 2B | 615365740 | 0.47 | -0.112 | 6.55 |  |
| CL_C_16 | CAP7_c16_483 | 2B | 777384902 | 1.10 | -0.085 | 5.49 | **TraesCS2B02G592400** (777375435-777385380)  GO:0004497 GO:0005506 GO:0009055 GO:0016705 GO:0020037 GO:0016021 GO:0044237 GO:0044238 GO:0044281 GO:0050896 GO:0055114  Cytochrome P450 superfamily IPR036396 |
| CL_C_BLUE | CAP7_c16_483 | 2B | 777384902 | 1.31 | -0.069 | 4.36 |  |
| CL_C_16 | IAAV6312 | 2D | 78765808 | 2.36 | 0.063 | 3.68 | **TraesCS2D02G133900** (78764128-78767414)  GO:0000462 GO:0006412 GO:0008152 GO:0003723 GO:0003735 GO:0005763  IPR000754 Ribosomal protein S9 |
| CL_C_BLUE | IAAV6312 | 2D | 78765808 | 2.05 | 0.055 | 3.49 |  |
| CL_S_16 | IAAV6312 | 2D | 78765808 | 1.97 | 0.189 | 7.55 |  |
| CL_S_17 | IAAV6312 | 2D | 78765808 | 0.89 | 0.064 | 3.68 |  |
| CL_S_BLUE | IAAV6312 | 2D | 78765808 | 2.21 | 0.086 | 4.98 |  |
| RSR_C_16 | BS00011109_51 | 2D | 409601404 | 0.51 | -0.050 | 3.50 | **TraesCS2D02G319200** (409599960-409604615)  GO:0005634 GO:0005737 GO:0016021 GO:0008168 GO:0016491 GO:0016043 GO:0016192 GO:0032259 GO:0032502 GO:0045184 GO:0046907 GO:0050789 GO:0055114  IPR025131 Domain of unknown function DUF4057 |
| RSR_C_18 | BS00011109_51 | 2D | 409601404 | -0.03 | -0.047 | 3.71 |  |
| RSR_C_BLUE | BS00011109_51 | 2D | 409601404 | -0.09 | -0.040 | 3.51 |  |
| RSR_C_17 | BobWhite_c2570_682 | 3A | 13739611 | -0.03 | -0.029 | 4.33 | **TraesCS3A02G026100** (13738823-13739563)  GO:0010467 GO:0034641 GO:0034645 GO:0044267 GO:0050789 |
| RSR_C_BLUE | BobWhite_c2570_682 | 3A | 13739611 | 0.15 | -0.035 | 5.71 |  |
| RSR_C_16 | Kukri_c18258_440 | 3A | 714425055 | 0.80 | 0.041 | 4.64 | **TraesCS3A02G485000** (714424957714430052)  [GO:0000166](http://amigo.geneontology.org/amigo/medial_search?q=GO:0000166) [GO:0003676](http://amigo.geneontology.org/amigo/medial_search?q=GO:0003676) [GO:0031072](http://amigo.geneontology.org/amigo/medial_search?q=GO:0031072) [GO:0003333](http://amigo.geneontology.org/amigo/medial_search?q=GO:0003333) [GO:0006396](http://amigo.geneontology.org/amigo/medial_search?q=GO:0006396) [GO:0006457](http://amigo.geneontology.org/amigo/medial_search?q=GO:0006457) [GO:0006952](http://amigo.geneontology.org/amigo/medial_search?q=GO:0006952) [GO:0017148](http://amigo.geneontology.org/amigo/medial_search?q=GO:0017148) [GO:0032501](http://amigo.geneontology.org/amigo/medial_search?q=GO:0032501) [GO:0044267](http://amigo.geneontology.org/amigo/medial_search?q=GO:0044267) [GO:0016021](http://amigo.geneontology.org/amigo/medial_search?q=GO:0016021) [GO:0019013](http://amigo.geneontology.org/amigo/medial_search?q=GO:0019013) [GO:1990904](http://amigo.geneontology.org/amigo/medial_search?q=GO:1990904)  IPR000504 RNA recognition motif domain |
| RSR_C_18 | Kukri_c18258_440 | 3A | 714425055 | 0.05 | 0.034 | 3.90 |  |
| RSR_C_BLUE | Kukri_c18258_440 | 3A | 714425055 | 0.20 | 0.038 | 5.51 |  |
| SL_TI_17 | Ex_c7626_444 | 4A | 16967638 | 4.06 | 0.015 | 3.00 | **TraesCS4A02G023700** (16964878-16968166)  [GO:0003723](http://amigo.geneontology.org/amigo/medial_search?q=GO:0003723) [GO:0006139](http://amigo.geneontology.org/amigo/medial_search?q=GO:0006139) [GO:0032502](http://amigo.geneontology.org/amigo/medial_search?q=GO:0032502) [GO:0044260](http://amigo.geneontology.org/amigo/medial_search?q=GO:0044260) [GO:0051641](http://amigo.geneontology.org/amigo/medial_search?q=GO:0051641) [GO:0065007](http://amigo.geneontology.org/amigo/medial_search?q=GO:0065007) [GO:0016021](http://amigo.geneontology.org/amigo/medial_search?q=GO:0016021)  IPR007656 GTD-binding domain |
| SL_TI_BLUE | Ex_c7626_444 | 4A | 16967638 | 2.26 | 0.013 | 3.91 |  |
| CL_C_16 | IAAV971 | 4B | 40752468 | 0.96 | -0.209 | 12.48 | **TraesCS4B02G051900** (40746325-40753688)  [GO:0004674](http://amigo.geneontology.org/amigo/medial_search?q=GO:0004674)[GO:0004713](http://amigo.geneontology.org/amigo/medial_search?q=GO:0004713) [GO:0005524](http://amigo.geneontology.org/amigo/medial_search?q=GO:0005524) [GO:0005634](http://amigo.geneontology.org/amigo/medial_search?q=GO:0005634) [GO:0005829](http://amigo.geneontology.org/amigo/medial_search?q=GO:0005829) [GO:0005856](http://amigo.geneontology.org/amigo/medial_search?q=GO:0005856)  [GO:0005886](http://amigo.geneontology.org/amigo/medial_search?q=GO:0005886) [GO:0012505](http://amigo.geneontology.org/amigo/medial_search?q=GO:0012505) [GO:0042995](http://amigo.geneontology.org/amigo/medial_search?q=GO:0042995) [GO:0032991](http://amigo.geneontology.org/amigo/medial_search?q=GO:0032991) [GO:0044422](http://amigo.geneontology.org/amigo/medial_search?q=GO:0044422) [GO:0006468](http://amigo.geneontology.org/amigo/medial_search?q=GO:0006468) [GO:0007165](http://amigo.geneontology.org/amigo/medial_search?q=GO:0007165) [GO:0032501](http://amigo.geneontology.org/amigo/medial_search?q=GO:0032501) [GO:0043687](http://amigo.geneontology.org/amigo/medial_search?q=GO:0043687) [GO:0008152](http://amigo.geneontology.org/amigo/medial_search?q=GO:0008152) [GO:0009987](http://amigo.geneontology.org/amigo/medial_search?q=GO:0009987) [GO:0032502](http://amigo.geneontology.org/amigo/medial_search?q=GO:0032502) [GO:0048856](http://amigo.geneontology.org/amigo/medial_search?q=GO:0048856)  IPR011009 Protein kinase-like domain superfamily |
| CL_C_17 | IAAV971 | 4B | 40752468 | 0.84 | -0.174 | 11.22 |  |
| CL_C_18 | IAAV971 | 4B | 40752468 | 1.78 | -0.143 | 10.71 |  |
| CL_C_BLUE | IAAV971 | 4B | 40752468 | 1.17 | -0.166 | 11.27 |  |
| CL_S_16 | IAAV971 | 4B | 40752468 | 1.32 | -0.203 | 6.83 |  |
| CL_S_17 | IAAV971 | 4B | 40752468 | 0.82 | -0.232 | 11.38 |  |
| CL_S_18 | IAAV971 | 4B | 40752468 | 3.40 | -0.124 | 4.75 |  |
| CL_S_BLUE | IAAV971 | 4B | 40752468 | 1.72 | -0.249 | 10.23 |  |
| RSR_C_16 | IAAV971 | 4B | 40752468 | 0.42 | 0.079 | 8.41 |  |
| RSR_C_17 | IAAV971 | 4B | 40752468 | -0.18 | 0.070 | 8.95 |  |
| RSR_C_18 | IAAV971 | 4B | 40752468 | 0.04 | 0.079 | 10.30 |  |
| RSR_C_BLUE | IAAV971 | 4B | 40752468 | -0.09 | 0.095 | 16.48 |  |
| RSR_S_16 | IAAV971 | 4B | 40752468 | 0.32 | 0.073 | 4.66 |  |
| RSR_S_BLUE | IAAV971 | 4B | 40752468 | 0.34 | 0.100 | 7.98 |  |
| SL_C_16 | IAAV971 | 4B | 40752468 | 0.94 | -0.621 | 7.38 |  |
| SL_C_17 | IAAV971 | 4B | 40752468 | 1.05 | -0.374 | 5.66 |  |
| SL_C_18 | IAAV971 | 4B | 40752468 | 0.60 | -0.454 | 6.60 |  |
| SL_C_BLUE | IAAV971 | 4B | 40752468 | 0.70 | -0.439 | 6.11 |  |
| CL_C_17 | Tdurum_contig42211_1634 | 4B | 97922966 | 0.72 | 0.094 | 5.53 | **TraesCS4B02G095100** (97921962-97925719)  GO:0005488 GO:0016788 GO:0005840 GO:0016021 GO:0006351 GO:0006355  IPR001810 F-box domain |
| CL_C_18 | Tdurum_contig42211_1634 | 4B | 97922966 | 2.40 | 0.083 | 6.25 |  |
| SL_S_17 | Kukri_c20822_1029 | 4B | 106973456 | 1.97 | 0.402 | 3.21 | **TraesCS4B02G101400** (106971617-106977017)  GO:0003006 GO:0006351 GO:0006355 GO:0009791 GO:0050896 GO:0003677 GO:0003700 GO:0005634  IPR003340 B3 DNA binding domain |
| SL_S_BLUE | Kukri_c20822_1029 | 4B | 106973456 | 0.18 | 0.378 | 3.07 |  |
| SL_C_16 | Kukri_rep_c68594_530 | 4D | 12773209 | 1.22 | -0.390 | 5.74 | **TraesCS4D02G029200** (12770045-12777226)  GO:0003723 GO:0004518 GO:0016740 GO:0005618 GO:0005635 GO:0005739 GO:0005783 GO:0005829 GO:0005856 GO:0005886 GO:0009506 GO:0009507 GO:0016442 GO:0035770 GO:0042470 GO:0006098 GO:0006351 GO:0006355 GO:0006740 GO:0009306 GO:0009651 GO:0009686 GO:0009845 GO:0022414 GO:0030244 GO:0031047 GO:0032787 GO:0046686 GO:0048193 GO:0051649 GO:0090305  IPR016685 RNA-induced silencing complex, nuclease component Tudor-SN |
| SL_C_17 | Kukri_rep_c68594_530 | 4D | 12773209 | 2.16 | -0.203 | 3.49 |  |
| SL_C_BLUE | Kukri_rep_c68594_530 | 4D | 12773209 | 1.20 | -0.237 | 3.78 |  |
| CL_S_17 | Kukri_c20631_614 | 4D | 358475408 | 0.63 | 0.068 | 3.09 | **TraesCS4D02G208200** (358475438-358480628 )  GO:0006464 GO:0034645 GO:0042157 GO:0008270 GO:0019706 GO:0016021  MF: palmitoyltransferase activity; protein-cysteine S-palmitoyltransferase activity  IPR001594 Palmitoyltransferase, DHHC domain |
| CL_S_BLUE | Kukri_c20631_614 | 4D | 358475408 | 1.33 | 0.069 | 3.55 |  |
| SL_C_17 | wsnp_Ex_c3620_6612231 | 5A | 2122622 | 1.42 | 0.356 | 7.76 | **TraesCS5A02G002700** (2120701-2126503)  GO:0000287 GO:0016757 GO:0050307 GO:0005634 GO:0005774 GO:0005829 GO:0005886 GO:0009506 GO:0016021 GO:0005986 GO:0016138 GO:0016311 GO:0032502 GO:0044260 GO:0046686 GO:0050794  IPR006380 Sucrose-phosphatase-like, N-terminal |
| SL_C_BLUE | wsnp_Ex_c3620_6612231 | 5A | 2122622 | 0.90 | 0.303 | 5.61 |  |
| SL_TI_17 | wsnp_JD_rep_c61843_39601402 | 5A | 41427412 | 2.68 | 0.021 | 3.23 | **TraesCS5A02G046100** (41423386-41427720)  [GO:0005085](http://amigo.geneontology.org/amigo/medial_search?q=GO:0005085) [GO:0008928](http://amigo.geneontology.org/amigo/medial_search?q=GO:0008928) [GO:0010471](http://amigo.geneontology.org/amigo/medial_search?q=GO:0010471) [GO:0010472](http://amigo.geneontology.org/amigo/medial_search?q=GO:0010472) [GO:0010473](http://amigo.geneontology.org/amigo/medial_search?q=GO:0010473) [GO:0010474](http://amigo.geneontology.org/amigo/medial_search?q=GO:0010474) [GO:0010475](http://amigo.geneontology.org/amigo/medial_search?q=GO:0010475) [GO:0030695](http://amigo.geneontology.org/amigo/medial_search?q=GO:0030695) [GO:0080046](http://amigo.geneontology.org/amigo/medial_search?q=GO:0080046) [GO:0080048](http://amigo.geneontology.org/amigo/medial_search?q=GO:0080048) [GO:0005634](http://amigo.geneontology.org/amigo/medial_search?q=GO:0005634) [GO:0005737](http://amigo.geneontology.org/amigo/medial_search?q=GO:0005737) [GO:0006006](http://amigo.geneontology.org/amigo/medial_search?q=GO:0006006) [GO:0006612](http://amigo.geneontology.org/amigo/medial_search?q=GO:0006612) [GO:0009408](http://amigo.geneontology.org/amigo/medial_search?q=GO:0009408) [GO:0009414](http://amigo.geneontology.org/amigo/medial_search?q=GO:0009414) [GO:0009611](http://amigo.geneontology.org/amigo/medial_search?q=GO:0009611) [GO:0009695](http://amigo.geneontology.org/amigo/medial_search?q=GO:0009695) [GO:0009723](http://amigo.geneontology.org/amigo/medial_search?q=GO:0009723) [GO:0009733](http://amigo.geneontology.org/amigo/medial_search?q=GO:0009733) [GO:0009738](http://amigo.geneontology.org/amigo/medial_search?q=GO:0009738) [GO:0009863](http://amigo.geneontology.org/amigo/medial_search?q=GO:0009863) [GO:0009867](http://amigo.geneontology.org/amigo/medial_search?q=GO:0009867) [GO:0010193](http://amigo.geneontology.org/amigo/medial_search?q=GO:0010193) [GO:0010363](http://amigo.geneontology.org/amigo/medial_search?q=GO:0010363) [GO:0019222](http://amigo.geneontology.org/amigo/medial_search?q=GO:0019222) [GO:0019853](http://amigo.geneontology.org/amigo/medial_search?q=GO:0019853) [GO:0034641](http://amigo.geneontology.org/amigo/medial_search?q=GO:0034641) [GO:0042538](http://amigo.geneontology.org/amigo/medial_search?q=GO:0042538) [GO:0042742](http://amigo.geneontology.org/amigo/medial_search?q=GO:0042742) [GO:0044260](http://amigo.geneontology.org/amigo/medial_search?q=GO:0044260) [GO:0050790](http://amigo.geneontology.org/amigo/medial_search?q=GO:0050790) [GO:0051704](http://amigo.geneontology.org/amigo/medial_search?q=GO:0051704) [GO:0052544](http://amigo.geneontology.org/amigo/medial_search?q=GO:0052544)  IPR026506 GDP-L-galactose/GDP-D-glucose phosphorylase |
| SL_TI_BLUE | wsnp_JD_rep_c61843_39601402 | 5A | 41427412 | 2.26 | 0.016 | 3.61 |  |
| SL_TI_17 | Ra_c69221_1167 | 5A | 41427501 | 2.68 | -0.021 | 3.23 |  |
| SL_TI_BLUE | Ra_c69221_1167 | 5A | 41427501 | 2.26 | -0.016 | 3.61 |  |
| SL_TI_17 | wsnp_Ra_rep_c69221_66574148 | 5A | 41427519 | 2.68 | -0.021 | 3.23 |  |
| SL_TI_BLUE | wsnp_Ra_rep_c69221_66574148 | 5A | 41427519 | 2.26 | -0.016 | 3.61 |  |
| SL_TI_17 | wsnp_Ra_rep_c69221_66574260 | 5A | 41427626 | 2.68 | 0.021 | 3.23 |  |
| SL_TI_BLUE | wsnp_Ra_rep_c69221_66574260 | 5A | 41427626 | 2.26 | 0.016 | 3.61 |  |
| SL_TI_17 | BS00031177_51 | 5A | 41427631 | 2.68 | -0.021 | 3.23 |  |
| SL_TI_BLUE | BS00031177_51 | 5A | 41427631 | 2.26 | -0.016 | 3.61 |  |
| RSR_C_16 | Tdurum_contig17712_200 | 5A | 535746207 | 0.46 | -0.056 | 3.76 | **TraesCS5A01G324800**(535746120-535746891) |
| RSR_C_18 | Tdurum_contig17712_200 | 5A | 535746207 | 0.00 | -0.046 | 4.03 |  |
| RSR_C_BLUE | Tdurum_contig17712_200 | 5A | 535746207 | 0.07 | -0.071 | 5.85 |  |
| CL_TI_18 | BS00082219_51 | 5A | 670346253 | 1.57 | -0.019 | 5.53 | **TraesCS5A02G504900** (670344832-670346541)  GO:0004190 GO:0005576 GO:0005840 GO:0016021 GO:0006508 GO:0006950 GO:0007275 GO:0009987 GO:0023052 GO:0008152 GO:0050794  IPR021109 Aspartic peptidase domain superfamily |
| CL_TI_BLUE | BS00082219_51 | 5A | 670346253 | 0.52 | -0.009 | 3.55 |  |
| CL_C_17 | Kukri_c75091_154 | 5A | 679657910 | 0.63 | -0.055 | 3.44 | **TraesCS5A02G516000** (679655516-679667091 )  GO:0003677 GO:0003725 GO:0004525 GO:0005524 GO:0008026 GO:0005634 GO:0005737 GO:0016442 GO:0006364 GO:0006397 GO:0007275 GO:0008033 GO:0009154 GO:0009207 GO:0016075 GO:0030422 GO:0035196 GO:0046034 GO:0046130 GO:0090502  IPR027417 P-loop containing nucleoside triphosphate hydrolase  IPR036085 PAZ domain superfamily  IPR036389 Ribonuclease III, endonuclease domain superfamily |
| CL_C_BLUE | Kukri_c75091_154 | 5A | 679657910 | 1.09 | -0.065 | 4.32 |  |
| RL_C_18 | Tdurum_contig65330_190 | 5B | 684615889 | -0.08 | 0.391 | 3.44 | **TraesCS5B02G523800** (684609906-684615867)  GO:0004222 GO:0005524 GO:0016887 GO:0005743 GO:0005886 GO:0009536 GO:0016021 GO:0006508 GO:0009154 GO:0009207 GO:0030163 GO:0046034 GO:0046130 GO:0051301  IPR037219 Peptidase M41-like  IPR027417 P-loop containing nucleoside triphosphate hydrolase |
| RL_C_BLUE | Tdurum_contig65330_190 | 5B | 684615889 | 0.80 | 0.331 | 3.34 |  |
| RSR_C_18 | Excalibur_c48387_58 | 5B | 684745996 | 0.35 | -0.035 | 3.17 | **TraesCS5B02G747400LC**  (684771622-684772574). |
| RSR_C_BLUE | Excalibur_c48387_58 | 5B | 684745996 | 0.28 | -0.034 | 4.43 |  |
| RSR_S_18 | D_GCE8AKX02IXEFJ_281 | 5D | 548869241 | 2.19 | 0.041 | 3.15 | scaffold19117-1 (547400205-552097092) |
| RSR_S_BLUE | D_GCE8AKX02IXEFJ_281 | 5D | 548869241 | 2.10 | 0.045 | 4.59 |  |
| SL_C_16 | wsnp_Ex_c35545_43677576 | 6A | 446301734 | 1.92 | 0.306 | 3.72 | **TraesCS6A02G236800** (446294149-446302176)  [GO:0000932](http://amigo.geneontology.org/amigo/medial_search?q=GO:0000932) [GO:0005829](http://amigo.geneontology.org/amigo/medial_search?q=GO:0005829) [GO:0010494](http://amigo.geneontology.org/amigo/medial_search?q=GO:0010494) GO:0003676 [GO:0005524](http://amigo.geneontology.org/amigo/medial_search?q=GO:0005524) [GO:0008026](http://amigo.geneontology.org/amigo/medial_search?q=GO:0008026) [GO:0006397](http://amigo.geneontology.org/amigo/medial_search?q=GO:0006397) [GO:0006413](http://amigo.geneontology.org/amigo/medial_search?q=GO:0006413) [GO:0006417](http://amigo.geneontology.org/amigo/medial_search?q=GO:0006417) [GO:0009154](http://amigo.geneontology.org/amigo/medial_search?q=GO:0009154) [GO:0009207](http://amigo.geneontology.org/amigo/medial_search?q=GO:0009207) [GO:0010501](http://amigo.geneontology.org/amigo/medial_search?q=GO:0010501) [GO:0019048](http://amigo.geneontology.org/amigo/medial_search?q=GO:0019048) [GO:0033962](http://amigo.geneontology.org/amigo/medial_search?q=GO:0033962) [GO:0044703](http://amigo.geneontology.org/amigo/medial_search?q=GO:0044703) [GO:0046034](http://amigo.geneontology.org/amigo/medial_search?q=GO:0046034) [GO:0046130](http://amigo.geneontology.org/amigo/medial_search?q=GO:0046130) [GO:0051028](http://amigo.geneontology.org/amigo/medial_search?q=GO:0051028)  IPR027417 P-loop containing nucleoside triphosphate hydrolase |
| SL_C_18 | wsnp_Ex_c35545_43677576 | 6A | 446301734 | 1.00 | 0.325 | 5.35 |  |
| SL_C_BLUE | wsnp_Ex_c35545_43677576 | 6A | 446301734 | 1.70 | 0.317 | 4.77 |  |
| RSR_TI_17 | Kukri_c37301_385 | 6A | 612178824 | 4.35 | -0.032 | 3.03 | **TraesCS6A02G407200** (612177309.-612180757)  [GO:0005488](http://amigo.geneontology.org/amigo/medial_search?q=GO:0005488) [GO:0005737](http://amigo.geneontology.org/amigo/medial_search?q=GO:0005737) [GO:0016607](http://amigo.geneontology.org/amigo/medial_search?q=GO:0016607) [GO:0006950](http://amigo.geneontology.org/amigo/medial_search?q=GO:0006950) [GO:0007186](http://amigo.geneontology.org/amigo/medial_search?q=GO:0007186) [GO:0009605](http://amigo.geneontology.org/amigo/medial_search?q=GO:0009605) [GO:0010099](http://amigo.geneontology.org/amigo/medial_search?q=GO:0010099) [GO:0019219](http://amigo.geneontology.org/amigo/medial_search?q=GO:0019219) [GO:0031326](http://amigo.geneontology.org/amigo/medial_search?q=GO:0031326) [GO:0035556](http://amigo.geneontology.org/amigo/medial_search?q=GO:0035556) [GO:0042221](http://amigo.geneontology.org/amigo/medial_search?q=GO:0042221) [GO:0044237](http://amigo.geneontology.org/amigo/medial_search?q=GO:0044237) [GO:0044238](http://amigo.geneontology.org/amigo/medial_search?q=GO:0044238) [GO:0048518](http://amigo.geneontology.org/amigo/medial_search?q=GO:0048518) [GO:0055121](http://amigo.geneontology.org/amigo/medial_search?q=GO:0055121) [GO:0080022](http://amigo.geneontology.org/amigo/medial_search?q=GO:0080022) [GO:1904667](http://amigo.geneontology.org/amigo/medial_search?q=GO:1904667)  IPR010756 Telomere length and silencing protein 1 |
| RSR_TI_BLUE | Kukri_c37301_385 | 6A | 612178824 | 1.98 | -0.023 | 3.13 |  |
| CL_C_16 | Kukri_c54773_76 | 6B | 41993574 | 0.97 | -0.116 | 3.17 | **TraesCS6B02G063300** (41991358-41994579)  [GO:0003922](http://amigo.geneontology.org/amigo/medial_search?q=GO:0003922) [GO:0004066](http://amigo.geneontology.org/amigo/medial_search?q=GO:0004066) [GO:0005524](http://amigo.geneontology.org/amigo/medial_search?q=GO:0005524) [GO:0016462](http://amigo.geneontology.org/amigo/medial_search?q=GO:0016462) [GO:0006177](http://amigo.geneontology.org/amigo/medial_search?q=GO:0006177) [GO:0006529](http://amigo.geneontology.org/amigo/medial_search?q=GO:0006529) [GO:0006541](http://amigo.geneontology.org/amigo/medial_search?q=GO:0006541) [GO:0034404](http://amigo.geneontology.org/amigo/medial_search?q=GO:0034404)  IPR017926 Glutamine amidotransferase  IPR001674 GMP synthase, C-terminal |
| CL_C_17 | Kukri_c54773_76 | 6B | 41993574 | 1.07 | -0.160 | 5.80 |  |
| CL_C_18 | Kukri_c54773_76 | 6B | 41993574 | 1.78 | -0.151 | 6.61 |  |
| RL_S_16 | wsnp_Ra_c33358_42248399 | 6B | 257993727 | 2.07 | -0.376 | 3.23 | **TraesCS6B02G205800** (257991181-257996938)  [GO:0000166](http://amigo.geneontology.org/amigo/medial_search?q=GO:0000166) [GO:0003723](http://amigo.geneontology.org/amigo/medial_search?q=GO:0003723) [GO:0003824](http://amigo.geneontology.org/amigo/medial_search?q=GO:0003824) [GO:0005654](http://amigo.geneontology.org/amigo/medial_search?q=GO:0005654) [GO:0005694](http://amigo.geneontology.org/amigo/medial_search?q=GO:0005694) [GO:0005856](http://amigo.geneontology.org/amigo/medial_search?q=GO:0005856) [GO:0016020](http://amigo.geneontology.org/amigo/medial_search?q=GO:0016020) [GO:1990904](http://amigo.geneontology.org/amigo/medial_search?q=GO:1990904) [GO:0042995](http://amigo.geneontology.org/amigo/medial_search?q=GO:0042995) [GO:0032991](http://amigo.geneontology.org/amigo/medial_search?q=GO:0032991) [GO:0044444](http://amigo.geneontology.org/amigo/medial_search?q=GO:0044444) [GO:1902494](http://amigo.geneontology.org/amigo/medial_search?q=GO:1902494) [GO:0006396](http://amigo.geneontology.org/amigo/medial_search?q=GO:0006396) [GO:0032501](http://amigo.geneontology.org/amigo/medial_search?q=GO:0032501) [GO:0009987](http://amigo.geneontology.org/amigo/medial_search?q=GO:0009987) [GO:0032502](http://amigo.geneontology.org/amigo/medial_search?q=GO:0032502) [GO:0048856](http://amigo.geneontology.org/amigo/medial_search?q=GO:0048856) [GO:0050794](http://amigo.geneontology.org/amigo/medial_search?q=GO:0050794)  IPR035979 RNA-binding domain superfamily |
| RL_S_BLUE | wsnp_Ra_c33358_42248399 | 6B | 257993727 | 0.43 | -0.294 | 3.36 |  |
| SL_C_18 | BobWhite_c22827_193 | 6B | 631740499 | 1.46 | 0.578 | 9.52 | **TraesCS6B02G359800** (631740171-631741196)  [GO:0006950](http://amigo.geneontology.org/amigo/medial_search?q=GO:0006950) [GO:0006996](http://amigo.geneontology.org/amigo/medial_search?q=GO:0006996) [GO:0009733](http://amigo.geneontology.org/amigo/medial_search?q=GO:0009733) [GO:0032502](http://amigo.geneontology.org/amigo/medial_search?q=GO:0032502) [GO:0044238](http://amigo.geneontology.org/amigo/medial_search?q=GO:0044238) [GO:0044260](http://amigo.geneontology.org/amigo/medial_search?q=GO:0044260) [GO:0050789](http://amigo.geneontology.org/amigo/medial_search?q=GO:0050789)  IPR003676 Small auxin-up RNA |
| SL_C_BLUE | BobWhite_c22827_193 | 6B | 631740499 | 2.00 | 0.351 | 3.82 |  |
| SL_C_17 | Tdurum_contig42489_768 | 6B | 718377799 | 1.71 | 0.323 | 4.87 | **TraesCS6B02G469900** (718375911-718381971)  [GO:0000151](http://amigo.geneontology.org/amigo/medial_search?q=GO:0000151) [GO:0005886](http://amigo.geneontology.org/amigo/medial_search?q=GO:0005886) [GO:0032991](http://amigo.geneontology.org/amigo/medial_search?q=GO:0032991) [GO:0004842](http://amigo.geneontology.org/amigo/medial_search?q=GO:0004842) [GO:0016881](http://amigo.geneontology.org/amigo/medial_search?q=GO:0016881) [GO:0006629](http://amigo.geneontology.org/amigo/medial_search?q=GO:0006629) [GO:0010115](http://amigo.geneontology.org/amigo/medial_search?q=GO:0010115) [GO:0010150](http://amigo.geneontology.org/amigo/medial_search?q=GO:0010150) [GO:0010260](http://amigo.geneontology.org/amigo/medial_search?q=GO:0010260) [GO:0010271](http://amigo.geneontology.org/amigo/medial_search?q=GO:0010271) [GO:0010380](http://amigo.geneontology.org/amigo/medial_search?q=GO:0010380) [GO:0016567](http://amigo.geneontology.org/amigo/medial_search?q=GO:0016567) [GO:0042180](http://amigo.geneontology.org/amigo/medial_search?q=GO:0042180) [GO:0044249](http://amigo.geneontology.org/amigo/medial_search?q=GO:0044249) [GO:0050896](http://amigo.geneontology.org/amigo/medial_search?q=GO:0050896)  IPR003613 U-box domain  Molecular Function. ubiquitin-protein transferase activity (GO:0004842) |
| SL_C_BLUE | Tdurum_contig42489_768 | 6B | 718377799 | 1.02 | 0.320 | 3.67 |  |
| CL_C_16 | RAC875_c25839_225 | 6D | 292066763 | 0.96 | -0.113 | 7.27 | **TraesCS6D02G206600** (292061396-292085830)  [GO:0000226](http://amigo.geneontology.org/amigo/medial_search?q=GO:0000226) [GO:0007049](http://amigo.geneontology.org/amigo/medial_search?q=GO:0007049) [GO:0009653](http://amigo.geneontology.org/amigo/medial_search?q=GO:0009653) [GO:0043242](http://amigo.geneontology.org/amigo/medial_search?q=GO:0043242) [GO:0043624](http://amigo.geneontology.org/amigo/medial_search?q=GO:0043624) [GO:0044238](http://amigo.geneontology.org/amigo/medial_search?q=GO:0044238) [GO:0051494](http://amigo.geneontology.org/amigo/medial_search?q=GO:0051494) [GO:0005694](http://amigo.geneontology.org/amigo/medial_search?q=GO:0005694) [GO:0005828](http://amigo.geneontology.org/amigo/medial_search?q=GO:0005828) [GO:0005881](http://amigo.geneontology.org/amigo/medial_search?q=GO:0005881) [GO:0005938](http://amigo.geneontology.org/amigo/medial_search?q=GO:0005938) [GO:0009524](http://amigo.geneontology.org/amigo/medial_search?q=GO:0009524) [GO:0016020](http://amigo.geneontology.org/amigo/medial_search?q=GO:0016020) [GO:0030054](http://amigo.geneontology.org/amigo/medial_search?q=GO:0030054) [GO:0044877](http://amigo.geneontology.org/amigo/medial_search?q=GO:0044877) [GO:0051010](http://amigo.geneontology.org/amigo/medial_search?q=GO:0051010)  IPR034085 TOG domain |
| CL_C_18 | RAC875_c25839_225 | 6D | 292066763 | 1.81 | -0.102 | 9.24 |  |
| RL_S_18 | Kukri_c860_353 | 7A | 513984676 | 0.90 | 0.518 | 3.52 | **TraesCS7A02G350700** (513983459-513991291)  GO:0003676 GO:0005524 GO:0008026 GO:0005739 GO:0009941 GO:0016021 GO:0009154 GO:0009207 GO:0010467 GO:0010501 GO:0046034 GO:0046130  IPR027417 P-loop containing nucleoside triphosphate hydrolase  IPR014001 Helicase superfamily 1/2, ATP-binding domain  IPR001650 Helicase, C-terminal |
| RL_S_BLUE | Kukri_c860_353 | 7A | 513984676 | 0.48 | 0.354 | 3.13 |  |
| RL_C_16 | wsnp_Ex_c52115_55827442 | 7A | 706832991 | 3.27 | 0.392 | 3.39 | **TraesCS7A02G746100LC** (706832099-706834157) no function |
| RL_C_BLUE | wsnp_Ex_c52115_55827442 | 7A | 706832991 | 0.81 | 0.321 | 3.99 |  |
| RL_C_16 | Excalibur_c52115_233 | 7A | 706833219 | 3.27 | -0.392 | 3.39 |  |
| RL_C_BLUE | Excalibur_c52115_233 | 7A | 706833219 | 0.81 | -0.321 | 3.99 |  |
| RL_C_16 | IAAV5828 | 7A | 706905999 | 3.25 | 0.393 | 3.33 | **TraesCS7A02G524600** (706905660-706912181)  [GO:0006633](http://amigo.geneontology.org/amigo/medial_search?q=GO:0006633) [GO:0008299](http://amigo.geneontology.org/amigo/medial_search?q=GO:0008299) [GO:0009414](http://amigo.geneontology.org/amigo/medial_search?q=GO:0009414) [GO:0010025](http://amigo.geneontology.org/amigo/medial_search?q=GO:0010025) [GO:0010143](http://amigo.geneontology.org/amigo/medial_search?q=GO:0010143) [GO:0010345](http://amigo.geneontology.org/amigo/medial_search?q=GO:0010345) [GO:0030433](http://amigo.geneontology.org/amigo/medial_search?q=GO:0030433) [GO:0032446](http://amigo.geneontology.org/amigo/medial_search?q=GO:0032446) [GO:0042335](http://amigo.geneontology.org/amigo/medial_search?q=GO:0042335) [GO:1900490](http://amigo.geneontology.org/amigo/medial_search?q=GO:1900490) [GO:0008270](http://amigo.geneontology.org/amigo/medial_search?q=GO:0008270) [GO:0061630](http://amigo.geneontology.org/amigo/medial_search?q=GO:0061630) [GO:0030176](http://amigo.geneontology.org/amigo/medial_search?q=GO:0030176)  IPR011016 Zinc finger, RING-CH-type  zinc ion binding |
| RL_C_BLUE | IAAV5828 | 7A | 706905999 | 0.81 | 0.319 | 3.82 |  |
| RL_C_16 | Kukri_c10757_183 | 7A | 706908576 | 3.27 | 0.390 | 3.30 |  |
| RL_C_BLUE | Kukri_c10757_183 | 7A | 706908576 | 0.84 | 0.316 | 3.79 |  |
| RL_C_16 | Excalibur_c1935_1762 | 7A | 706908857 | 3.23 | 0.401 | 3.02 |  |
| RL_C_BLUE | Excalibur_c1935_1762 | 7A | 706908857 | 0.80 | 0.308 | 3.13 |  |
| RL_C_16 | IAAV1543 | 7A | 706909286 | 3.27 | 0.390 | 3.30 |  |
| RL_C_BLUE | IAAV1543 | 7A | 706909286 | 0.84 | 0.316 | 3.79 |  |
| RL_C_16 | BobWhite_c24096_57 | 7A | 706910751 | 3.25 | 0.393 | 3.33 |  |
| RL_C_BLUE | BobWhite_c24096_57 | 7A | 706910751 | 0.82 | 0.319 | 3.82 |  |
| CL_C_17 | Excalibur_c46453_144 | 7A | 719568332 | 0.90 | 0.068 | 4.38 | **TraesCS7A02G542900** (719566911-719570377)  [GO:0005768](http://amigo.geneontology.org/amigo/medial_search?q=GO:0005768) [GO:0005802](http://amigo.geneontology.org/amigo/medial_search?q=GO:0005802) [GO:0016021](http://amigo.geneontology.org/amigo/medial_search?q=GO:0016021) [GO:0008270](http://amigo.geneontology.org/amigo/medial_search?q=GO:0008270) [GO:0016757](http://amigo.geneontology.org/amigo/medial_search?q=GO:0016757) [GO:0009987](http://amigo.geneontology.org/amigo/medial_search?q=GO:0009987) [GO:0022414](http://amigo.geneontology.org/amigo/medial_search?q=GO:0022414) [GO:0032501](http://amigo.geneontology.org/amigo/medial_search?q=GO:0032501) [GO:0044238](http://amigo.geneontology.org/amigo/medial_search?q=GO:0044238)  IPR006852 Protein of unknown function DUF616 |
| CL_C_BLUE | Excalibur_c46453_144 | 7A | 719568332 | 1.36 | 0.070 | 3.62 |  |
| CL_S_18 | BS00068033_51 | 7A | 721417500 | 3.32 | 0.088 | 4.20 | **TraesCS7A02G545300** (721411549-721417543 )  [GO:0007275](http://amigo.geneontology.org/amigo/medial_search?q=GO:0007275) [GO:0009891](http://amigo.geneontology.org/amigo/medial_search?q=GO:0009891) [GO:0016070](http://amigo.geneontology.org/amigo/medial_search?q=GO:0016070) [GO:0019219](http://amigo.geneontology.org/amigo/medial_search?q=GO:0019219) [GO:0044249](http://amigo.geneontology.org/amigo/medial_search?q=GO:0044249) [GO:0050896](http://amigo.geneontology.org/amigo/medial_search?q=GO:0050896)  auxin response factor 2 |
| CL_S_BLUE | BS00068033_51 | 7A | 721417500 | 2.16 | 0.075 | 3.37 |  |
| SL_TI_17 | BS00068033_51 | 7A | 721417500 | 2.82 | -0.017 | 3.44 |  |
| SL_TI_BLUE | BS00068033_51 | 7A | 721417500 | 2.48 | -0.012 | 3.19 |  |
| SL_TI_17 | BS00068032_51 | 7A | 721417546 | 2.85 | -0.016 | 3.30 |  |
| SL_TI_BLUE | BS00068032_51 | 7A | 721417546 | 2.47 | -0.012 | 3.41 |  |
| SL_TI_17 | BS00061911_51 | 7A | 722573912 | 3.42 | 0.021 | 5.44 | scaffold65961 (722365381-723353682) |
| SL_TI_BLUE | BS00061911_51 | 7A | 722573912 | 3.36 | 0.013 | 3.90 |  |
| RL_C_17 | Ku_c884_2297 | 7B | 44623174 | 3.43 | -0.149 | 4.37 | **TraesCS7B02G045600** (44622961-44626647)  GO:0002764 GO:0006468 GO:0007275 GO:0009581 GO:0009626 GO:0010204 GO:0010359 GO:0016045 GO:0016567 GO:0032940 GO:0042742 GO:0008152 GO:0004674 GO:0005524 GO:0005886 GO:0009507 GO:0016021 GO:0031410  IPR003591 Leucine-rich repeat, typical subtype  IPR000719 Protein kinase domain |
| RL_C_BLUE | Ku_c884_2297 | 7B | 44623174 | 1.37 | -0.187 | 3.14 |  |
| RL_C_18 | BS00064344_51 | 7B | 44878503 | -0.06 | 0.238 | 3.39 | scaffold67584 (36860267-58773506) |
| RL_C_BLUE | BS00064344_51 | 7B | 44878503 | 1.10 | 0.200 | 3.24 |  |
| RL_S_18 | Kukri_rep_c72909_657 | 7B | 46351517 | 1.45 | 0.430 | 4.09 | **TraesCS7B02G047100** (46343243-46351992 )  [GO:0003677](http://amigo.geneontology.org/amigo/medial_search?q=GO:0003677) [GO:0046982](http://amigo.geneontology.org/amigo/medial_search?q=GO:0046982) [GO:0060090](http://amigo.geneontology.org/amigo/medial_search?q=GO:0060090) [GO:0005634](http://amigo.geneontology.org/amigo/medial_search?q=GO:0005634) [GO:0016021](http://amigo.geneontology.org/amigo/medial_search?q=GO:0016021) [GO:0008152](http://amigo.geneontology.org/amigo/medial_search?q=GO:0008152) [GO:0009987](http://amigo.geneontology.org/amigo/medial_search?q=GO:0009987) [GO:0019219](http://amigo.geneontology.org/amigo/medial_search?q=GO:0019219) [GO:0048467](http://amigo.geneontology.org/amigo/medial_search?q=GO:0048467) [GO:0048513](http://amigo.geneontology.org/amigo/medial_search?q=GO:0048513)  IPR029005 LIM-domain binding protein/SEUSS |
| RL_S_BLUE | Kukri_rep_c72909_657 | 7B | 46351517 | 1.33 | 0.267 | 3.05 |  |
| RL_TI_18 | Kukri_rep_c72909_657 | 7B | 46351517 | 0.80 | 0.016 | 3.82 |  |
| RL_TI_BLUE | Kukri_rep_c72909_657 | 7B | 46351517 | 2.47 | 0.009 | 3.27 |  |
| RL_C_18 | BS00089942_51 | 7B | 626239239 | 0.08 | 0.263 | 4.35 | **TraesCS7B02G363900** (626238481-626242957)  [GO:0004842](http://amigo.geneontology.org/amigo/medial_search?q=GO:0004842) [GO:0008270](http://amigo.geneontology.org/amigo/medial_search?q=GO:0008270) [GO:0005737](http://amigo.geneontology.org/amigo/medial_search?q=GO:0005737) [GO:0016021](http://amigo.geneontology.org/amigo/medial_search?q=GO:0016021) [GO:0006573](http://amigo.geneontology.org/amigo/medial_search?q=GO:0006573) [GO:0009083](http://amigo.geneontology.org/amigo/medial_search?q=GO:0009083) [GO:0032446](http://amigo.geneontology.org/amigo/medial_search?q=GO:0032446) [GO:0044257](http://amigo.geneontology.org/amigo/medial_search?q=GO:0044257) [GO:0050789](http://amigo.geneontology.org/amigo/medial_search?q=GO:0050789) [GO:0055114](http://amigo.geneontology.org/amigo/medial_search?q=GO:0055114) [GO:1901606](http://amigo.geneontology.org/amigo/medial_search?q=GO:1901606)  IPR011016 Zinc finger, RING-CH-type |
| RL_C_BLUE | BS00089942_51 | 7B | 626239239 | 0.77 | 0.192 | 3.23 |  |
| SL_TI_16 | Kukri_rep_c79716_287 | 7B | 718467281 | 0.65 | 0.022 | 2.99 | **TraesCS7B02G461100** (718465552-718471588)  [GO:0003677](http://amigo.geneontology.org/amigo/medial_search?q=GO:0003677) [GO:0004402](http://amigo.geneontology.org/amigo/medial_search?q=GO:0004402) [GO:0005634](http://amigo.geneontology.org/amigo/medial_search?q=GO:0005634) [GO:0009536](http://amigo.geneontology.org/amigo/medial_search?q=GO:0009536) [GO:0016021](http://amigo.geneontology.org/amigo/medial_search?q=GO:0016021) [GO:0006355](http://amigo.geneontology.org/amigo/medial_search?q=GO:0006355) [GO:0006366](http://amigo.geneontology.org/amigo/medial_search?q=GO:0006366) [GO:0016573](http://amigo.geneontology.org/amigo/medial_search?q=GO:0016573) [GO:0008152](http://amigo.geneontology.org/amigo/medial_search?q=GO:0008152) [GO:0051276](http://amigo.geneontology.org/amigo/medial_search?q=GO:0051276)  IPR036427 Bromodomain-like superfamily |
| SL_TI_BLUE | Kukri_rep_c79716_287 | 7B | 718467281 | 2.25 | 0.015 | 3.17 |  |
| SL_TI_16 | Kukri_rep_c79716_389 | 7B | 718467383 | 0.65 | -0.022 | 2.99 |  |
| SL_TI_BLUE | Kukri_rep_c79716_389 | 7B | 718467383 | 2.25 | -0.015 | 3.17 |  |
| SL_TI_16 | BS00096151_51 | 7B | 718471358 | 0.65 | 0.022 | 2.99 |  |
| SL_TI_BLUE | BS00096151_51 | 7B | 718471358 | 2.25 | 0.015 | 3.17 |  |

**Control (C), Stress (S), Coleoptile length (CL), Shoot length (SL), Root Length (RL), Root/Shoot ratio (RSR), Tolerance index (TI), Chr: Chromosome; Position (Physical, pb); -log_10_ (p-value (SNP)).**
