# Supplementary material for: Association mapping unravels the genetics controlling seedling drought stress tolerance in winter wheat
Source: Front Plant Sci. 2023 Feb 2;14:1061845. doi: 10.3389/fpls.2023.1061845 (PMC9933780; doi:10.3389/fpls.2023.1061845)
Supplement: Supplementary file 2 [file DataSheet_1.pdf]

TraesCS1B02G480400 (687794252..687799812)1B

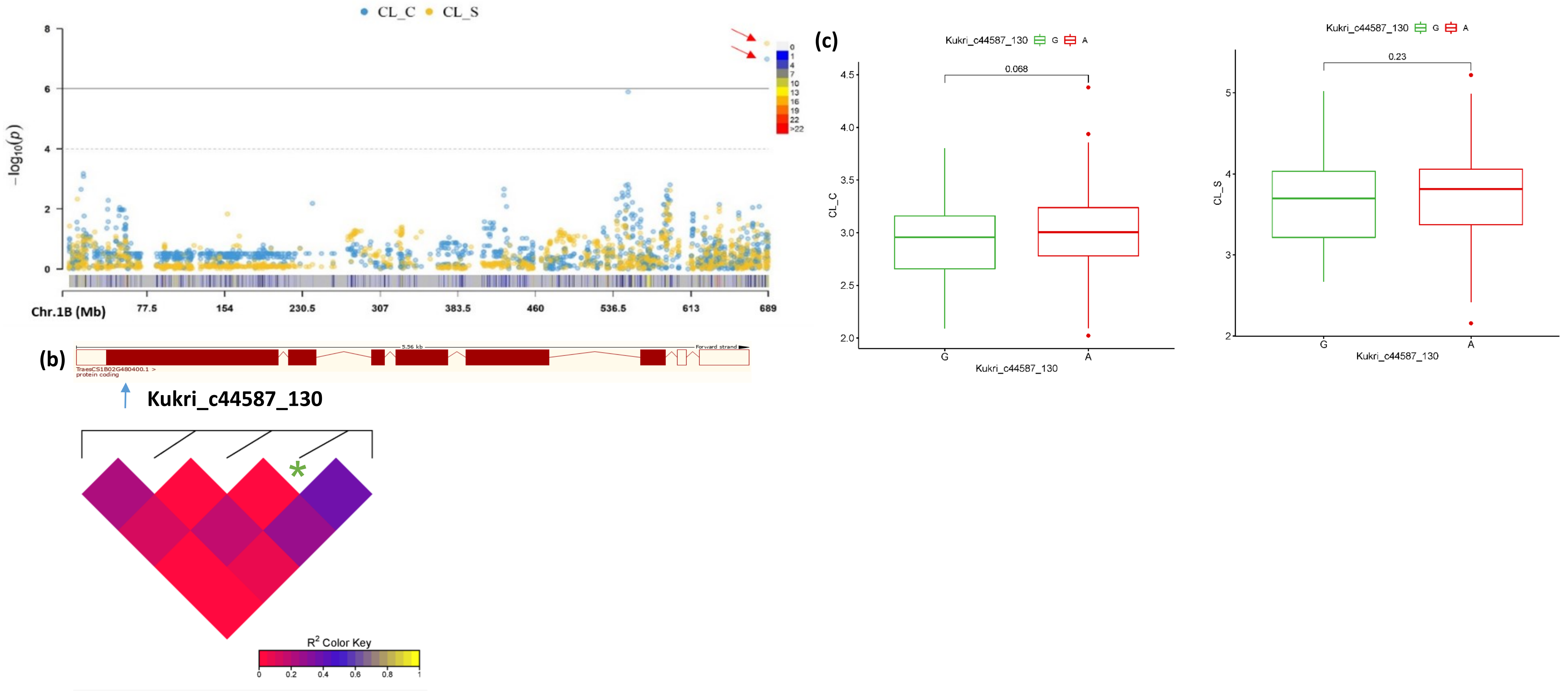

Figure S1. (a) Manhattan plots showing significant marker traits association for 261 winter wheat genotypes used for seedling growth parameters under (C) control and (S) drought stress (CL_C and CL_S): CL: coleoptile length. Multitraits MTAs are indicated as (P<0.001; -log10> 3). (b) the structure of the *TraesCS1B02G480400* gene with the position of the co-located QTN (687794252..687799812)1B within the gene and the linkage disequilibrium (LD) interval and (C) QTN -gene haplotype analysis.

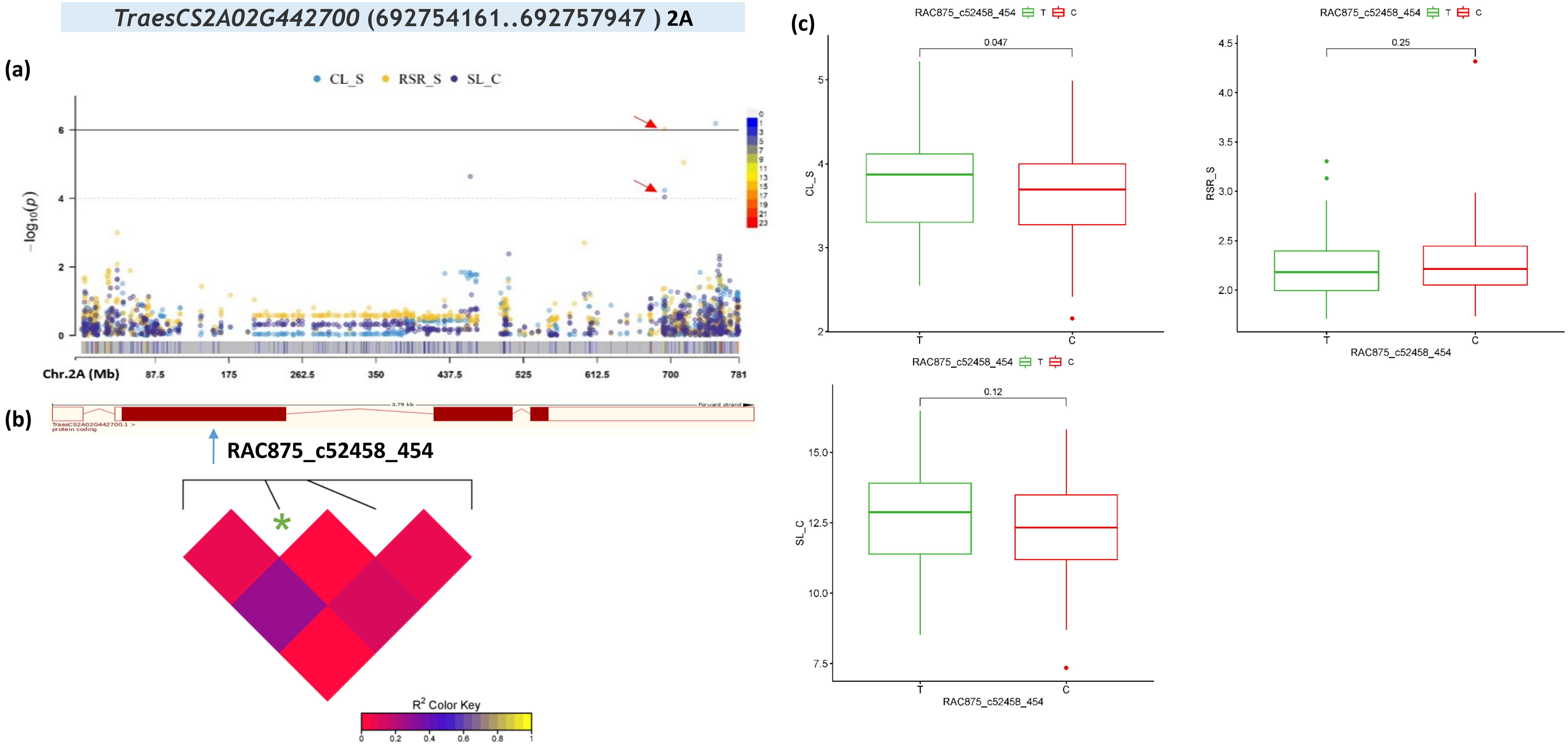

Figure S2. (a) Manhattan plots showing significant marker traits association for 261 winter wheat genotypes used for seedling growth parameters under (C) control and (S) drought stress (CL_S, RSR_S, and SL_C): CL: coleoptile length, RSR: root to shoot ratio, SL: shoot length. Multitraits MTAs are indicated as (P<0.001; -log10> 3). (b) the structure of the *TraesCS2A02G442700* gene with the position of the co-located QTN (692754161..692757947 bp)2A within the gene and the linkage disequilibrium (LD) interval and (C) QTN -gene haplotype analysis.

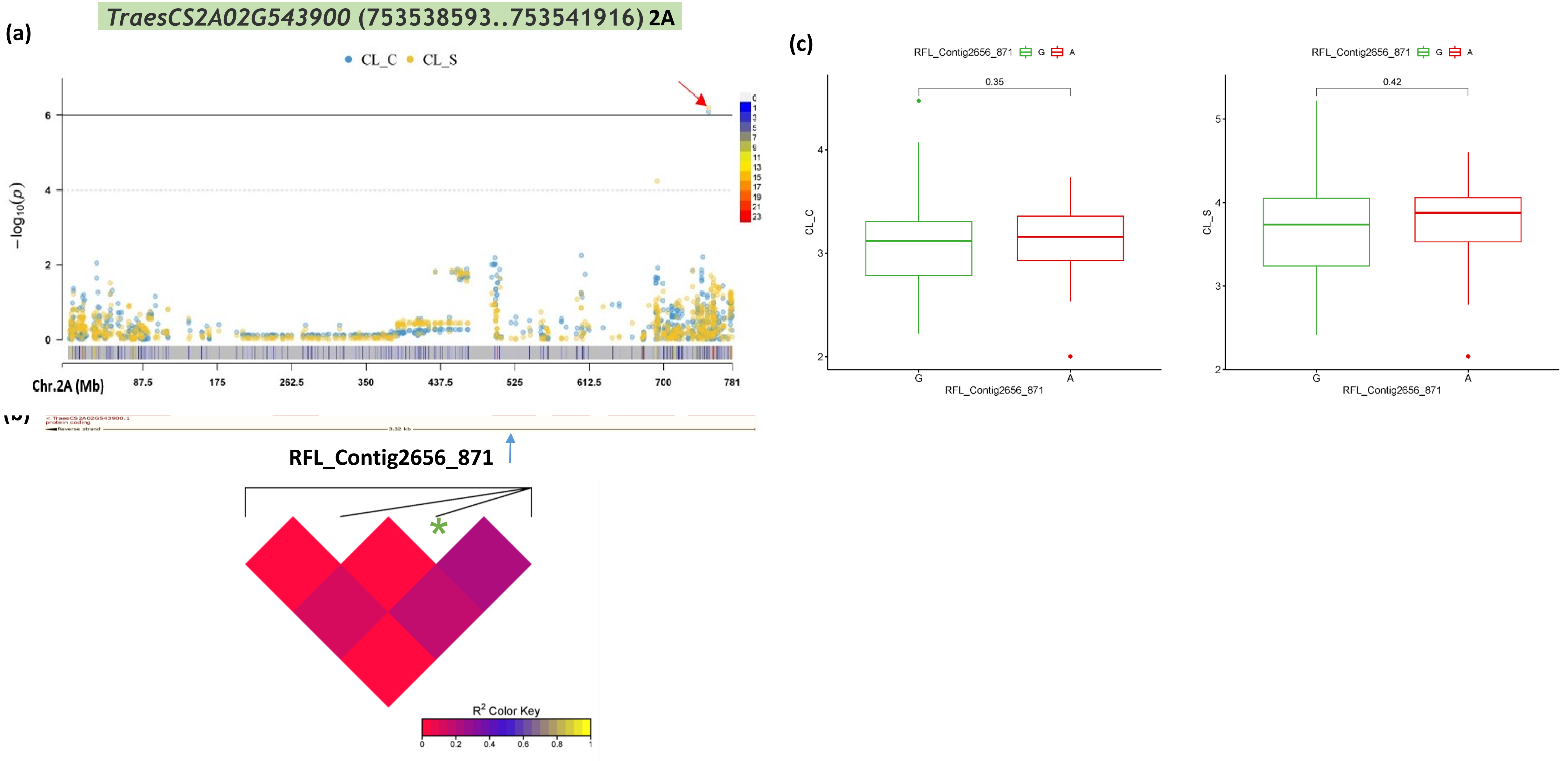

Figure S3. (a) Manhattan plots showing significant marker traits association for 261 winter wheat genotypes used for seedling growth parameters under (C) control and (S) drought stress (CL_C and CL_S): CL: coleoptile length. Multitraits MTAs are indicated as (P<0.001; -log10> 3). (b) the structure of the *TraesCS2A02G543900* gene with the position of the co-located QTN (753538593..753541916) 2A within the gene and the linkage disequilibrium (LD) interval and (C) QTN -gene haplotype analysis.

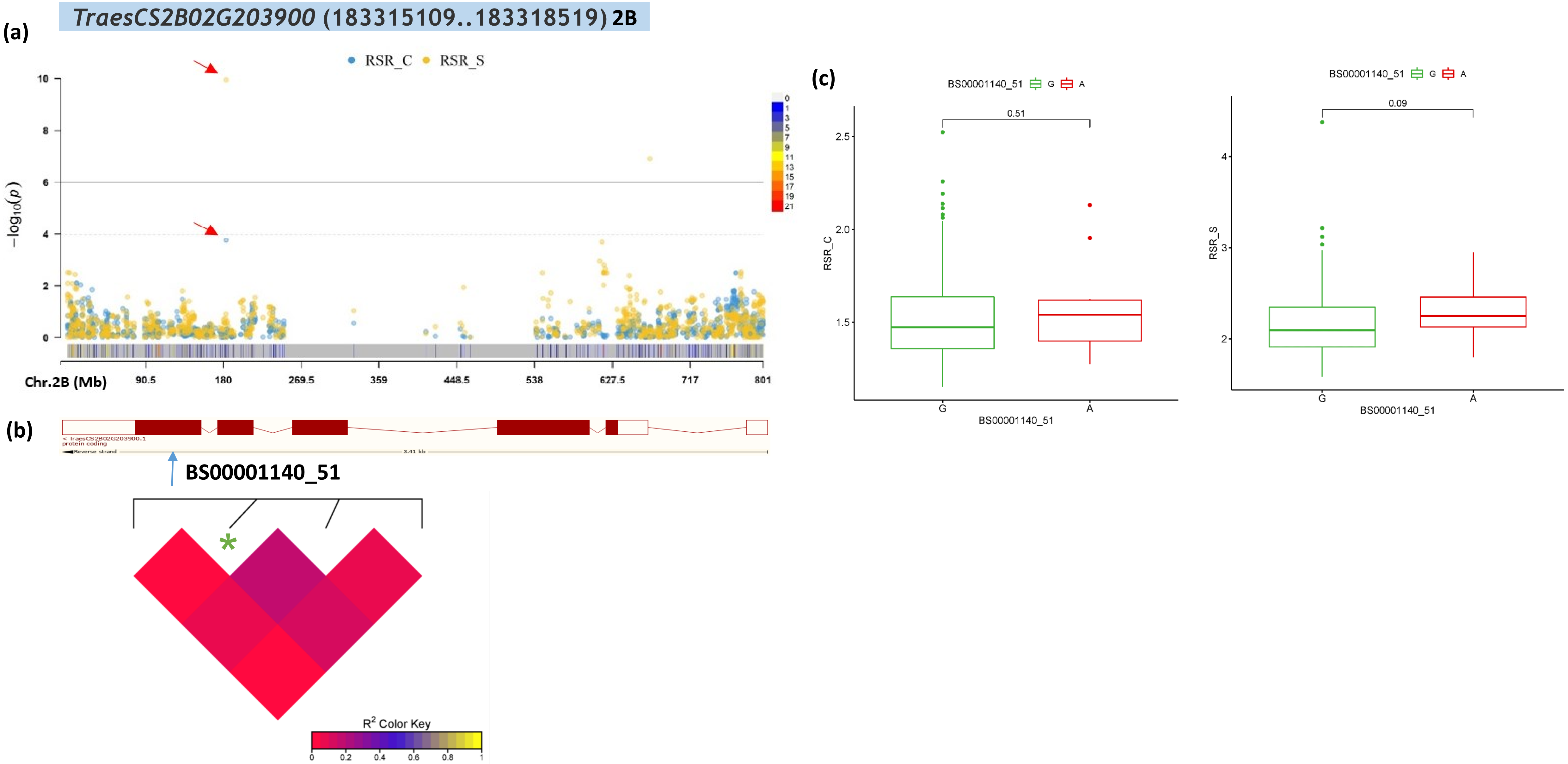

Figure S4. (a) Manhattan plots showing significant marker traits association for 261 winter wheat genotypes used for seedling growth parameters under (C) control and (S) drought stress (RSR_C and RSR_S): RSR: root to shoot ratio. Multitraits MTAs are indicated as (P<0.001; -log10> 3). (b) the structure of the *TraesCS2B02G203900* gene with the position of the co-located QTN (183315109..183318519) 2B within the gene and the linkage disequilibrium (LD) interval and (C) QTN -gene haplotype analysis.

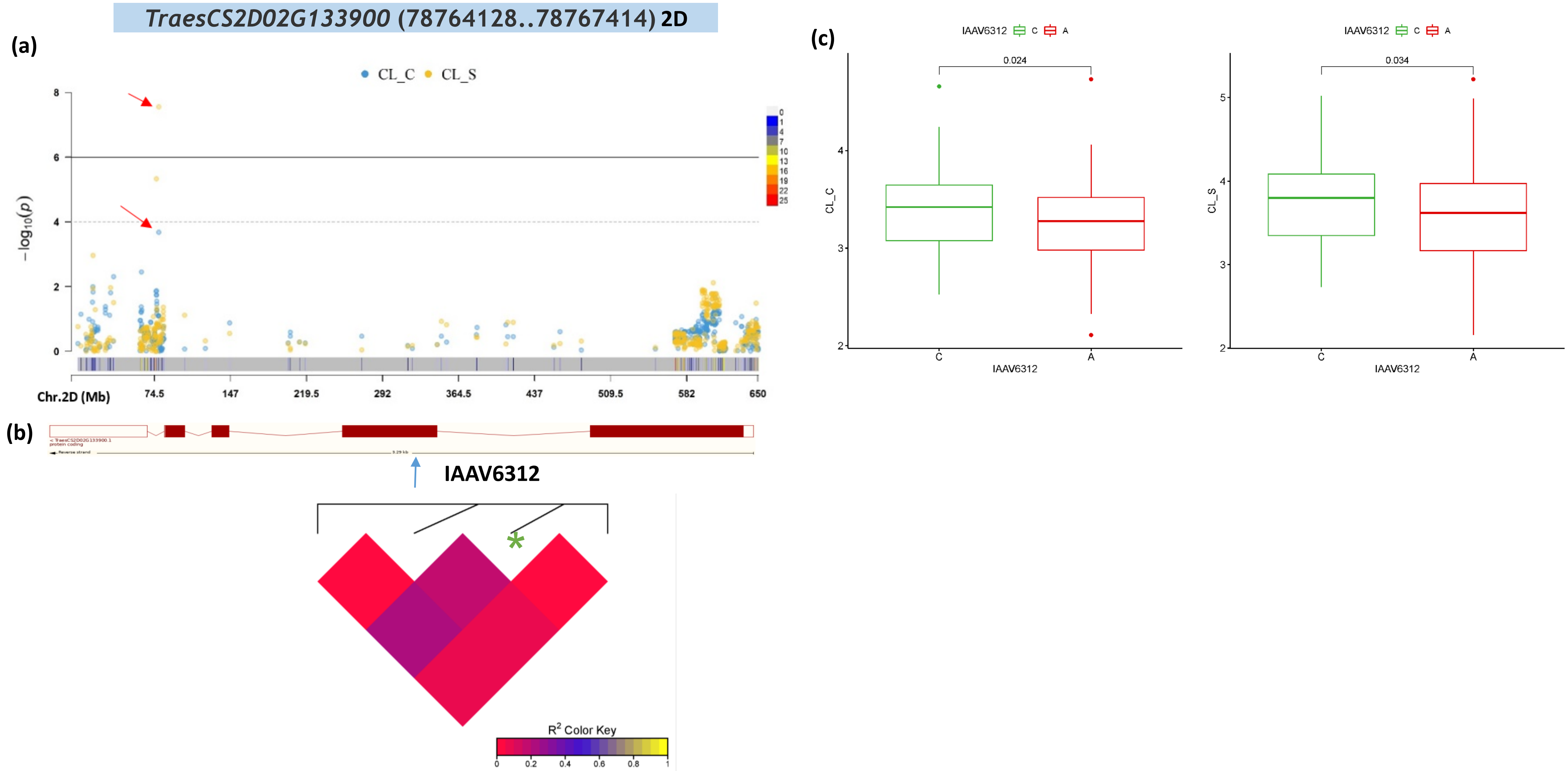

Figure S5. (a) Manhattan plots showing significant marker traits association for 261 winter wheat genotypes used for seedling growth parameters under (C) control and (S) drought stress (CL_C and CL_S): CL: coleoptile length. Multitraits MTAs are indicated as (P<0.001; -log10> 3). (b) the structure of the *TraesCS2D02G133900* gene with the position of the co-located QTN (78764128..78767414) 2D within the gene and the linkage disequilibrium (LD) interval and (C) QTN -gene haplotype analysis.

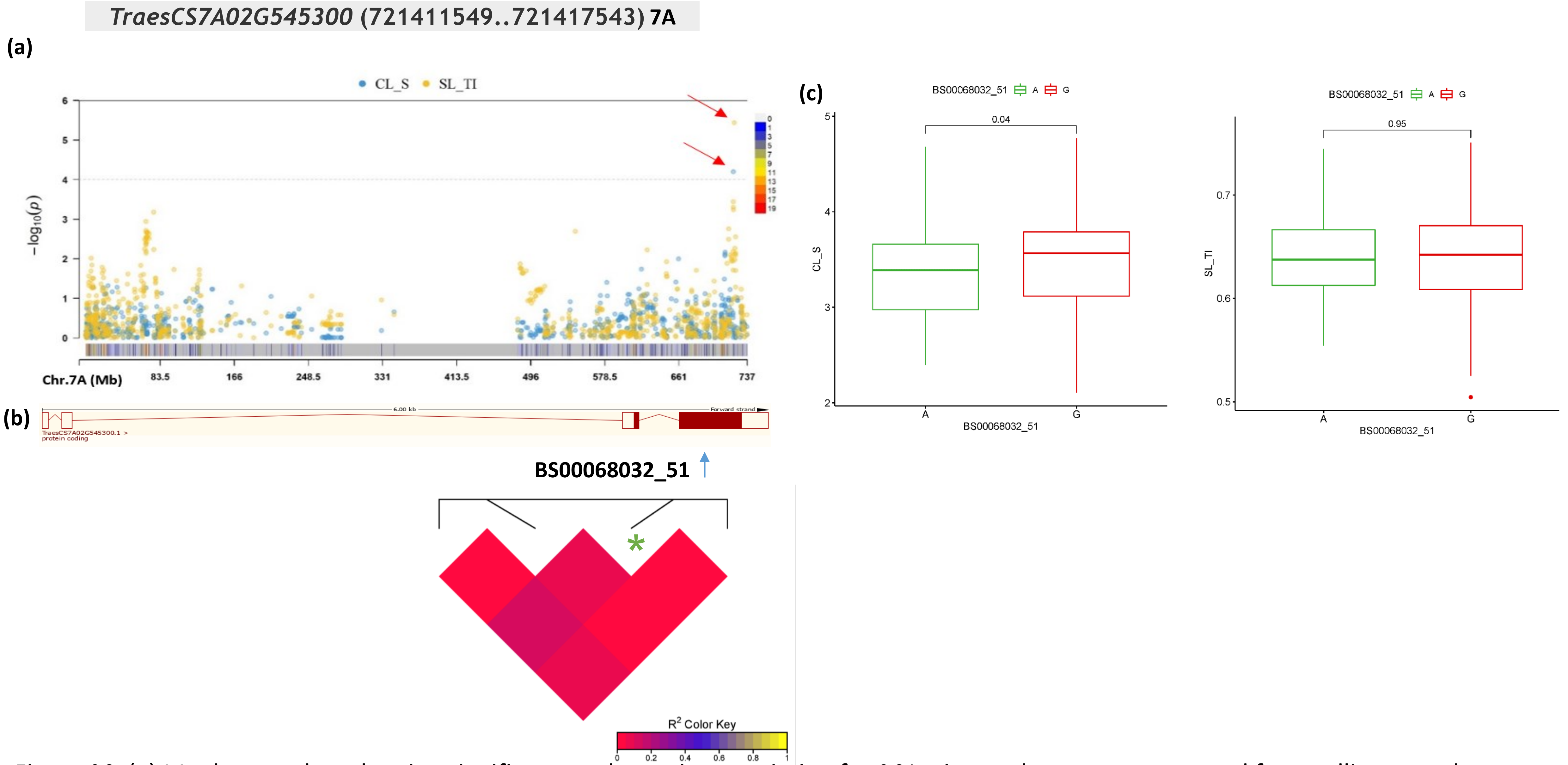

Figure S6. (a) Manhattan plots showing significant marker traits association for 261 winter wheat genotypes used for seedling growth parameters under (C) control and (S) drought stress (CL_S and SL_TI): CL: coleoptile length, SL: shoot length, and TI: tolerance index. Multitraits MTAs are indicated as (P<0.001; -log10> 3). (b) the structure of the *TraesCS7A02G545300* gene with the position of the co-located QTN (721411549..721417543) 7A within the gene and the linkage disequilibrium (LD) interval and (C) QTN -gene haplotype analysis.

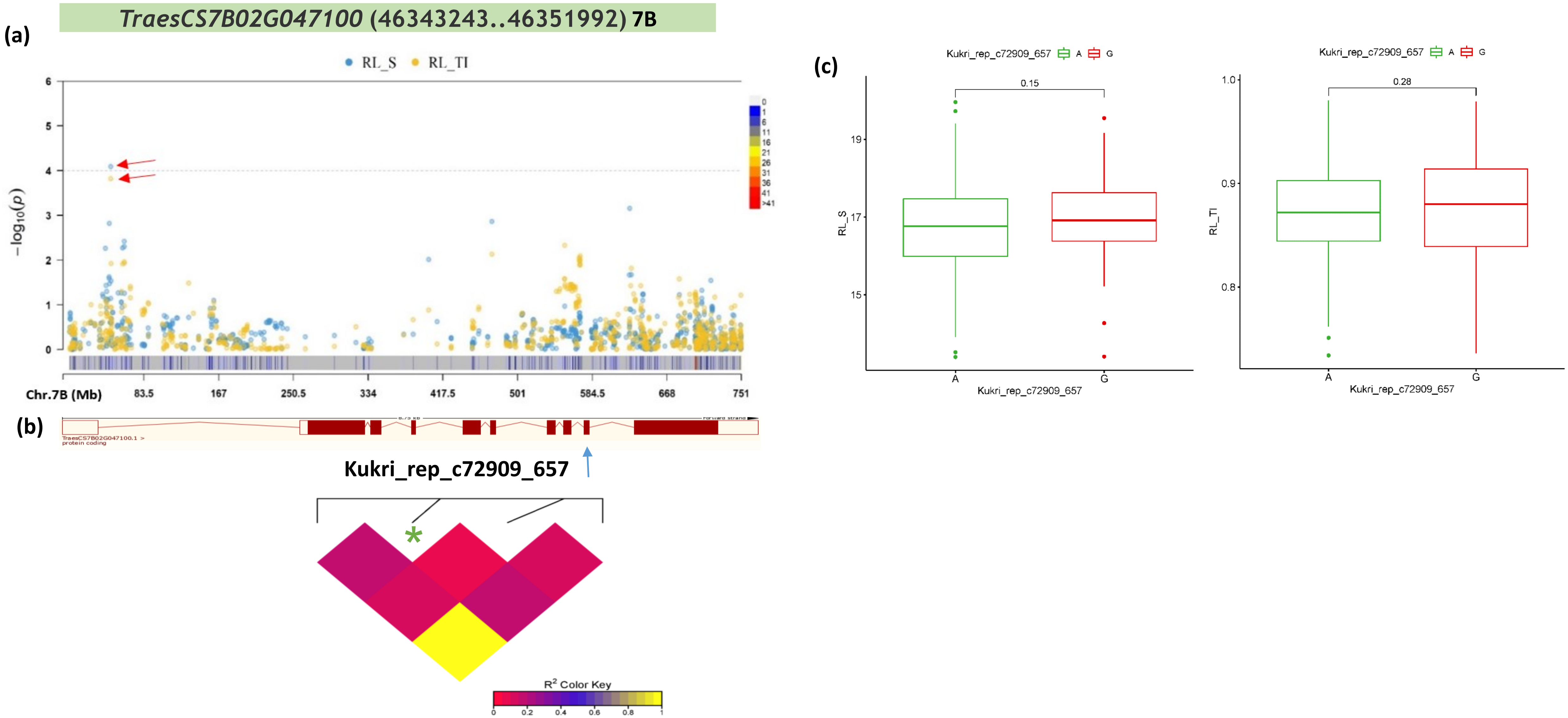

Figure S7. (a) Manhattan plots showing significant marker traits association for 261 winter wheat genotypes used for seedling growth parameters under (C) control and (S) drought stress (RL_S and RL_TI): RL: root length, and TI: tolerance index. Multitraits MTAs are indicated as (P<0.001; -log10> 3). (b) the structure of the *TraesCS7B02G047100* gene with the position of the co-located QTN (46343243..46351992) 7B within the gene and the linkage disequilibrium (LD) interval and (C) QTN -gene haplotype analysis.
